# The RAAS system SNPs polymorphism is associated with essential hypertension risk in rural areas in northern China

**DOI:** 10.7150/ijms.98724

**Published:** 2024-10-14

**Authors:** Jin Cheng, Jing Cui, Yuanyuan Li, Xiaona Liu, Yuting Jiang, Qiaoling Liu, Chang Liu, Hongqi Feng, Zhe Jiao, Xinhua Shao, Yanhui Gao, Dianjun Sun, Wei Zhang

**Affiliations:** 1 Center for Endemic Disease Control, Chinese Center for Disease Control and Prevention, Harbin Medical University, Harbin, People's Republic of China.; 2 National Health Commission & Education Bureau of Heilongjiang Province, Key Laboratory of Etiology and Epidemiology, Harbin Medical University (23618504), Harbin, People's Republic of China.; 3 Heilongjiang Provincial Key Laboratory of Trace Elements and Human Health, Harbin Medical University, Harbin, People's Republic of China.; 4 Center for Chronic Disease Prevention and Control, Harbin Medical University, Harbin, People's Republic of China.; 5 Harbin Center for Disease Control and Prevention, Harbin, Heilongjiang, People's Republic of China.; 6 State Key Laboratory of Frigid Zone Cardiovascular Diseases (SKLFZCD), Harbin Medical University, Harbin, People's Republic of China.

**Keywords:** Hypertension, RAAS system, SNP, Gene score

## Abstract

**Objectives:** Epidemiological evidence has shown that genetics and environment are associated with the risk of hypertension. However, the specific SNP effects of a cluster of crucial genes in the RAAS system on the risk of hypertension are unclear.

**Methods:** A case-control study was performed on the baseline participants of Environment and Chronic Disease in Rural Areas of Heilongjiang China (ECDRAHC) study. According to the inclusion and exclusion criteria, 757 subjects (428 hypertensive patients) were enrolled. A total of 32 SNP sites and related haplotypes, involved in *AGT (angiotensinogen)*, *ACE (angiotensin-converting enzyme)*, *AGTR1*, *CYP11B2 (aldosterone-synthase)*,* LDLR (low-density lipoprotein receptor)*, *LRP5 (low-density lipoprotein receptor associated protein 5)*, *LRP6 (low-density lipoprotein receptor associated protein 6)*, *PPARG (peroxisome proliferator-activated receptor gamma)* and *ACE2 (angiotensin-converting enzyme 2)* genes which exert important roles in renin-angiotensin-aldosterone system (RAAS) system were analyzed. Furthermore, a polygenic scoring model was established to assess individual risk of developing hypertension based on the comprehensive SNPs effects in genes related the RAAS system.

**Results:** After controlling the impact of confounding factors, multivariate logistic regression analysis revealed that the distribution of* AGT/rs5046*, *LRP6/rs12823243* and *ACE2/rs2285666* was associated with susceptibility to essential hypertension. In genetic score model, the score > -0.225 had a higher risk, the *OR* (*95%CI)* was 1.229 (1.110, 1.362).

**Conclusions:** To the best of our knowledge, this is the first time a hypertension risk scoring model on RAAS associated gene cluster has been constructed, which will provide a novel approach for prevention and control of essential hypertension.

## Introduction

Hypertension, referred to as a silent killer, is recognized as the leading modifiable risk factor for the global burden of cardiovascular diseases (CVD), characterized by high incidence rate, high disability rate and high mortality 1. As reported in *The Lancet* by the NCD Risk Factor Collaboration (NCD-RFC), the global number of hypertensive patients exceeded one billion in 2019, which represents a doubling since 1990. Furthermore, hypertension was directly responsible for 10.8 million fatalities 2-4. A recent comprehensive population survey revealed that around 245 million Chinese adults suffer from hypertension in 2019 5,6. In addition, there are significant differences in the epidemiological distribution of hypertension in different regions of China, and the prevalence rate is higher in northeast China 7, 8.

Hypertension is a complex multi-system disease, associated with genetics and environment, involving the kidney, cardiovascular system and central nervous system. The prevalence of hypertension increased in correlation with higher BMI and older age, as well as variables like salt consumption, excessive alcohol use, and other recognized risk factors, including experience of cold exposure 9-14. Individuals with lower levels of education or residing in rural areas appeared increased susceptibility as a consequence of an unhealthy lifestyle with a high-sodium diet and insufficient heating in winter 15, 16.

Accumulating evidence demonstrates over 1,477 SNPs associated with blood pressure 17-21, and the RAAS system is predominantly involved 22. The RAAS system is an important regulatory system for maintaining stable blood pressure, mainly including REN (renin), ACE, AGT, CYP11B2, ACE2 23-25. In classic RAAS system, AGT is associated with the generation of angiotensin II (Ang II), Ang II activates its Angiotensin II type 1 (AT1) receptor, the main receptor mediating vasoconstriction, aldosterone secretion, and renal sodium (Na^+^) re-absorption, which increased blood pressure and promoted the development of hypertension 26,27. ACE, in addition to its potent constrictive effect on blood vessels, also promotes aldosterone secretion 28. Some studies have reported that receptors related to the RAAS system are involved in blood pressure regulation, such as trans-membrane proteins LDLR, LRP5, and LRP6 29. ACE2 protects against excessive activation of AT1 in the heart tissues. Additionally, renal *ACE2* was down-regulated in three different models of hypertension 30,31. Although the SNP does not directly alter the coding sequence, it possibly influences the gene function by disrupting the transcription activity of genes. Studies generally concentrate on the impact of the SNP in individual gene or nearby genes associated with RAAS system on the pathogenesis of hypertension, and the complete effects of the SNPs on a cluster gene in the RAAS system are uncertain 26-31.

Heilongjiang province is located in the remote northeastern region of China and is renowned for its extended and frigid winters, with average outdoor temperature dropping below -14°C in the winter. The prevalence rate of essential hypertension (EH) in Heilongjiang is remarkably higher than the national average level. The purpose of this study is to investigate the association between the different SNPs of a cluster of genes in RAAS system and hypertension, based on ECDRAHC cohort study 32,33. Furthermore, we evaluate each individual risk of developing hypertension by calculating a polygenic score based on the comprehensive impacts of a cluster gene linked to the RAAS system. Deciphering these associations may help in identifying effective strategies for preventing and managing hypertension in chilly regions of China.

## Materials and Methods

### Study design and participants

The ECDRAHC is a prospective cohort study on the environment and chronic disease in rural areas of Heilongjiang, China, covering nine regions of Ming shui County 34. The baseline survey of the ECDRAHC cohort was conducted from November 2018 to September 2019. The research participants were selected from a total of 1682 adults, aged, who were Han nationality and residing in Tongda Town. ① Case group: the subjects were included based on 2018 Chinese Guidelines for the Prevention and Treatment of Hypertension. We selected only one patient among the survey subjects who suffered from hypertension in first-step relatives. ② Control group: subjects with blood pressure levels<140/90 mmHg [systolic blood pressure < 140 mmHg and/or diastolic blood pressure < 90 mmHg, (HBP, systolic blood pressure ≥140 mmHg and/or diastolic blood pressure ≥90 mmHg)] 35. Based on the above inclusion criteria and exclusion criteria (details of exclusion criteria are showed in the Supplement), 757 subjects were divided into two groups.

For assessment of environmental factors, data were collected from the questionnaires, physical measurements and laboratory testing of blood samples. Physicians utilize internationally standardized electronic blood pressure monitors to measure blood pressure accurately. A total of 25 biochemical indicators, including ALP, ALT, AST, GGT, CREA, GLU, TCH, etc., were measured using the Hitachi 3100 automatic biochemical analyzer (More details of criterion for the environmental factors and biochemical measures are showed in Table S1).

The blood samples were collected by medical professionals according to strict aseptic procedures. Approximately 5 mL of fasting (over 8 hours) peripheral venous blood of the subjects was put into vacuum collection tubes with ethylene diamine tetraacetic acid (EDTA) anticoagulants and regular vacuum collection tubes, respectively. Serum samples were separated and centrifuged at 3000 r/min for 10 min after a little standing. The blood samples were stored at -20℃ on site after being divided, and stored in the -80℃ refrigerator when they were returned to the laboratory on the same day.

### Analysis of genotype

Through reviewing the literatures on RAAS system gene polymorphisms and cardiovascular diseases, specific SNP loci were chosen. Next, the SNP loci were evaluated by Genesky Biotechnologies Inc., Shanghai, including region (gene region), population (Han Chinese in Beijing or Southern Han Chinese), minor allele frequency (if >0.01) and R^2^ (if >0.8) of linkage disequilibrium analysis. Eventually, 34 eligible SNPs (including *ACE2*) were included in the analysis. The TIANamp Blood DNA Kit was used to extract human genomic DNA, and all DNA samples were store at -80℃. The genotyping was detected by the multiple SNP typing kit (Genesky, Shanghai, China), and the analyses were entrusted to Genesky laboratory. STREGA quality criteria: the concentration of DNA samples should be above 10 ng/μL, the total amounts of DNA should be greater than 200 ng, the absorbance ratio (A260/A280) should be greater than 1.80, and genomic banding should be complete and clear without any degradation. Primers were designed and aligned using primer 5 software and referring to NCBI online primer design tool (primer-blast, http://www.ncbi.nlm.nih.gov/tools/primer-blast) and NCBI-Basic Local Alignment Search Tool.

### Statistical analysis

All statistical analyses were conducted using IBM SPSS Statistics 25.0 (Chicago, IL, USA). If the data follows a normal distribution, the mean ± SD and N (%) represent continuous variables and categorical variables, respectively. If the data does not conform to a normal distribution, median (IQR) is used to describe the data distribution. Differences in demographic and biochemical characteristics were analyzed using chi-square test, student's test for categorical and continuous variables, respectively. Chi-square test was employed to explore whether the SNP sites of the control samples were consistent with Hardy-Weinberg Equilibrium (HWE). Chi-square test and Fisher exact probability method were applied to explore the difference in allele frequency and genotype frequency in SNP sites for each target gene. Logistic regression analysis was conducted to investigate the association of gene polymorphism and possible impact factors with hypertension. Haploview software (ver 4.2) was employed to analyze linkage disequilibrium (LD) and haplotype construction, with adjacent sites R^2^ (>0.3) and D' (>0.8) as the judgement standards for linkage disequilibrium. *P*<0.05 was considered statistically significant.

### Genetic Score (or Allele Score)

Genetic Score is a valid causal estimate of the overall effects of multiple genetic variants on a multifactorial disease. In this investigation all gene loci were taken into account to calculate a weighted gene score for each subject, with reference to some excellent literature 36. The weighted gene score is calculated by multiplying the number of mutant alleles at each SNP site by their respective weights, and then summing these products. The formula for calculating the weighted gene score is as follows:



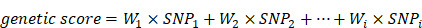



In the above formula, “i” represents the number of SNPs that constitute the weighted gene score; “W” is the weight corresponding to each SNP site (the weight of the SNP site is the regression coefficient β of each SNP site calculated by logistic regression analysis); “SNP” represents the number of minor alleles in each SNP site, denoted by unweighted scores: “0, 1, 2” (For example, genotype AA = 0, Aa = 1, aa = 2).

## Results

### Demographic characteristics and serum index results

A total of 428 participants (223 women) were essential hypertensive patients, and 329 subjects entered the control group according to the inclusion and exclusion criteria. The study found that individuals with essential hypertension were elderly and exhibited higher levels of BMI, ALP, GGT, CREA, GLU, TCH, TG, LDL, ApoA1, ApoB, and LDH (Table 1). Significant variations were observed in waist circumference, BMI, ALP, GLU, TG, LDL, ApoA1, ApoB, LDH and vegetables consumption among female hypertension participants (Table S2). In addition, the levels of waist circumference, ALP, CU exhibited a significant increase in male patients (Table S3).

### Risk factors for essential hypertension

The collinearity diagnosis indicated that there was no collinearity between various risk factors for all subjects (Table S4). According to the standard of *P* < 0.1, thirteen risk factors were screened, including age, waist circumference, BMI, ALP, CREA, GLU, TG, LDL, ApoA1, ApoB, UA, CA and CU (Table 2). With the exception of CREA, there were significant differences among female hypertensive patients in term of age, waist circumference, BMI, ALP, GLU, TG, LDL, ApoA1, ApoB, UA, CA and CU (*p* < 0.05). However, only levels of waist circumference, ALP and CU increased significantly in male patients (*p* < 0.05). (Table S5, Table S6).

### Hardy-Weinberg equilibrium test

We explored 32 SNP polymorphic sites (except *ACE2*) in the present study, including *AGT* (rs699, rs2493134, rs2004776, rs2148582, rs5046, rs3789679), *ACE* (rs4316, rs4343, rs4461142), *AGTR1* (rs5182, rs1492100, rs5186, rs275646, rs2933249, rs2638360), *CYP11B2*(rs6433, rs3802228, rs1799998), *LDLR*/rs688,* LRP5* (rs638051, rs556442), *LRP6* (rs10743980, rs11054731, rs2417086, rs7136900, rs12823243), *PPARG* (rs3856806, rs1175543, rs2972164, rs13433696, rs9817428, rs12631819), all sites passed the H-W test (p > 0.05), and had MAF greater than 5%. Due to the distribution of the *ACE2* gene on the X chromosome, only the analysis for female populations in the controls was performed. See more details in Table S7.

### Comparison of the allele frequency distribution in the case and control group

The differences in allele frequency distribution of *LRP5/rs638051* and *PPARG/rs3856806* are statistically significant between the case and control group (*p* < 0.05). However, no obvious difference was observed in the distribution of other loci (*p* > 0.05). See more details in Table S8.

### Distribution and risk assessment of EH for each genotype

After controlling for the impact of 13 risk factors listed in Table 2, we analyzed the association between the genotypes of 32 SNP polymorphic sites and hypertension. The logistic regression results showed that the distribution of the *AGT/rs5046* codominant genetic model, *LRP6/rs12823243* codominant genetic model, and recessive genetic model were statistically different between the cases and controls (*p* < 0.05). The distribution of *AGT/rs5046* and *LRP6/rs12823243* was statistically different between the cases and controls (*p* < 0.05), and OR (95% CI) was [5.49 (1.08, 27.97), genotype AA] and [0.01 (0.01, 0.30), genotype TT], respectively. The results of *AGT* and *LPR6* are shown in Table 3, and other sites are shown in Table S9.

### Comparison of the *ACE2* gene allele frequency distribution and risk assessment of EH for each genotype

The genotype distribution of *ACE2* SNP site between the EH case group and the control group is shown in Table 4. The multivariate logistic regression results showed that the genotype distribution of *rs2285666* was statistically different between the cases and controls in males (*p* < 0.05). The OR (95%CI) of codominant genetic model was [1.70(1.02, 2.83), genotype CC], dominant genetic model was [1.69(1.05,2.71), genotype (CT+CC)], and recessive genetic model was [1.79(1.11,2.90), genotype CC]. Among females, the OR (95%CI) of *rs2285666* codominant genetic model was [1.76(1.02, 3.03), genotype CT] and the dominant genetic model was 1.69(1.02, 2.80), genotype (CT+CC)]. These results were statistically significant (p < 0.05).

### The gene haplotype construction and its association with EH

**Figure 1:**
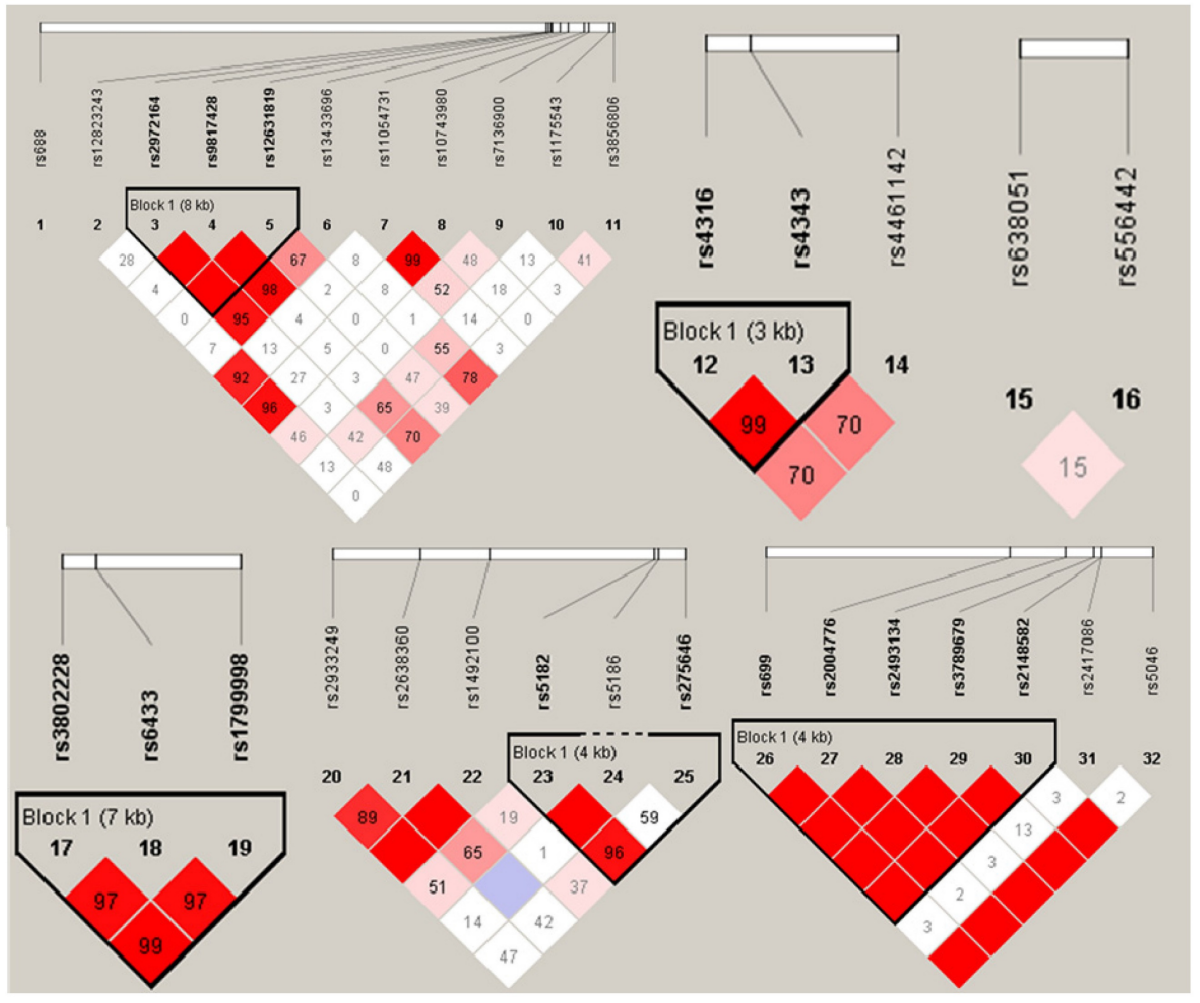
Haplogroups and haplotype distribution maps.

Haploview 4.2 software was utilized to construct haplotype and analyze the linkage disequilibrium (LD), the results were shown in Figure 1. D' value >0.8 was set as the criteria of strong linkage relationship. The results showed that seven haploid blocks were constructed in 32 SNP sites, and 25 haplotypes were taken as independent variable to analyze the correlation with hypertension. The results showed that none of the haplotype distribution was statistically different in EH case group and control group after controlling the influence of 13 impact factors as mentioned before (*p* > 0.05, Table 5).

### Results of genetic score

A total of 32 SNP sites (excluding *ACE2* genes) were weighted to obtain the genetic score of RAAS system genes as a whole with the occurrence of EH. As was shown in Table 6, the median score for the overall population was -0.225. Based on this median score the genetic scores of each study subject were divided into two groups: high score group (score > -0.225), and low score group (score ≤ -0.225). The result of regression model showed that the OR (95%CI) of high score group was 1.229 (1.110-1.362), *p* < 0.01. Based on the above results, AGT/rs5046 and LRP6/rs12823243 were significantly correlated with the occurrence of EH. Additionally, we developed a two-gene scoring model, however, it failed to demonstrate a significant rise in risk for EH between the high score group and the low score group. Therefore, the results suggested that the genetic score for whole genes model could predict the risk of EH (more details in Table 6). Furthermore, the age was divided into two time periods according to less than 59 and more than 60, and whether they were EH patients as an outcome variable. In Table 7, the log-rank test was then conducted on the genetic score model. The findings indicated that there was a remarkable difference in the hazard function curves between the two groups, the OR and 95%CI of high score group were 2.150 (1.998-2.377) (*p* < 0.01). In Table 8, the result of stratification analysis between genetic score and age indicated that genetic score > -0.225 and age ≥ 60 were associated with increased susceptibility to hypertension (*p* < 0.05).

## Discussion

This study observed that the SNP scoring of a cluster gene in the RAAS system are positively associated with essential hypertension. First, our gene score modeling incorporated several a cluster of genes related to the RAAS system for the first time. Second, we explored the association between the SNPs, haplotypes of the RAAS system related genes and essential hypertension, and we acquired some significant discoveries. Third, we combined comprehensive effects of environmental factors with SNPs on the occurrence of essential hypertension.

In the current study, *AGT* genotype AA at locus *rs5046* was found to have a greater risk on EH. A previous study had obtained similar results, Qian Li et al. investigated the susceptibility to EH in isolated populations (Yi and Hani) in remote areas of Yunnan, China, and found that *rs5046* was significantly associated with EH in the Hani ethnic group 37. However, other studies got different results, a Mexican group published the role of *AGT* gene cluster (*rs699, rs4762, rs5051, rs5049, rs5046*) polymorphisms in coronary artery disease, but no meaningful findings were found for the* rs5046* locus 38. The TT genotype at the *LRP6/rs12823243* locus was found to be a risk factor for the occurrence of hypertension in the present study. A previous study has shown similar findings that carriers of *LRP6* mutations was associated with cardiovascular disease 39. Another investigation conducted in in the United States revealed that people with mutations in the *LRP6* gene had higher-than-normal serum-related markers, which may be associated with the development of essential hypertension 40. We found that the CC genotype at the *ACE2/rs285666* locus was a risk factor for EH in males and the TT genotype in females, respectively. Consistent with our findings, Yan He et al. found that the T allele or TT genotype of *ACE2/rs2285666* was a risk factor for hypertension in Wa women 41. *ACE2* is a critical component of RAAS that is located on the X chromosome. Therefore, the escape of X inactivation may contribute to the sex-related differences in the *ACE2* gene in hypertension 41-43.

Genetic risk score is able to superimpose the micro-effects of each polymorphic locus to improve the accuracy of disease risk prediction. In the established gene scoring model, we included all the loci (except *ACE2*), and accounted for variations in alleles at each locus. Subsequently, we evaluated the cumulative risk of grouping genetic score by log rank test. Our finding indicated that scores over -0.225 are associated with a higher risk. When the score was above -0.225, the cumulative risk of EH increased by 2.150 times. Moreover, researchers have modeled different forms of gene scoring to measure the effect of changes in a single allele in a cluster of genes on the risk of disease, mostly for the prediction of cancer prognosis 44, 45. Nonetheless, a limitation of the current study is the absence of data on gene expression levels, which would have facilitated the development of a more comprehensive scoring system.

Furthermore, LDH, CU, and GGT were risk factors for cardiovascular disease in previous studies 46-48. However, we only found a significant difference in copper ions between EH cases and controls in the present study, the discrepancies may be mainly attributed to genetic background.

## Conclusions

Collectively, this is the first time a hypertension risk scoring model of RAAS gene cluster has been constructed, which will provide novel insights for the prevention and control of chronic cardiovascular diseases such as essential hypertension. In addition, we found genetic polymorphisms of *AGT/rs5046*, *LPR6/rs12823243*, and *ACE2/rs2285666* were associated with essential hypertension in residents of the cold regions of China.

## Supplementary Material

Supplementary information and tables.

## Figures and Tables

**Table 1:** Demographic characteristics and serum index determination for all subjects

Characteristics		Control (n = 329)	Case (n = 428)	*T /χ^2^*	*P-value*
BP					
SBP, mean±SD (mmHg)		122.56 ± 9.91	153.47 ± 17.56	62.010	< 0.001
DBP, mean±SD (mmHg)		80.02 ± 6.65	95.36 ± 10.99	33.350	< 0.001
Sociodemographic factors					
Age, mean±SD (year)		52.98 ± 8.73	57.90 ± 7.64	2.855	< 0.001
Sex, N (%)	Female	194 (58.97)	223 (52.10)	3.542	0.060
	Male	135 (41.03)	205 (47.90)		
Marital status, N (%)	Unmarried	22 (6.69)	39 (9.11)	1.477	0.224
	Married	307 (93.31)	389 (90.89)		
Nine-year compulsory education, N (%)	Uncompleted	290 (88.15)	375 (87.62)	0.049	0.825
	Completed	39 (11.85)	53 (12.38)		
Lifestyle behaviors					
Current smoking, N (%)	No	187 (56.84)	263 (61.45)	1.640	0.200
	Yes	142 (43.16)	165 (38.55)		
Passive smoking, N (%)	No	195 (59.27)	244 (57.01)	0.390	0.532
	Yes	134 (40.73)	184 (42.99)		
Harmful drinking, N (%)	No	264 (80.24)	332 (77.57)	0.794	0.373
	Yes	65 (19.76)	96 (22.43)		
Physical exertion, N (%)	Light	210 (63.83)	263 (61.45)	1.825	0.401
	Medium	18 (5.47)	34 (7.94)		
	Heavy	101 (30.70)	131 (30.61)		
Exercise regularly, N (%)	No	228 (69.30)	297 (69.39)	0.001	0.978
	Yes	101 (30.70)	131 (30.61)		
Adiposity measures					
Waist, mean ± SD (cm)		84.64 ± 8.45	88.85 ± 9.36	0.336	< 0.001
Hip, mean ± SD (cm)		97.23 ± 7.83	99.97 ± 7.89	0.054	< 0.001
WHR, mean ± SD		0.87 ± 0.11	0.89 ± 0.10	0.018	0.023
BMI, mean ± SD		24.26 ± 2.89	25.62 ± 3.39	4.645	< 0.001
Medical history					
Family history of Hypertension, N (%)	No	267 (81.16)	346 (80.84)	0.012	0.913
	Yes	62 (18.84)	82 (19.16)		
Family history of Diabetes, N (%)	No	311 (94.53)	413 (96.50)	1.725	0.189
	Yes	18 (5.47)	15 (3.50)		
Family history of Cardiovascular disease, N (%)	No	309 (93.92)	403 (94.16)	0.019	0.891
	Yes	20 (6.08)	25 (5.84)		
Clinical and biochemical measures					
ALP, mean±SD (U/L)		79.99 ± 24.44	89.54 ± 29.44	5.834	< 0.001
ALT, mean±SD (U/L)		22.30 ± 16.29	23.23 ± 13.56	0.441	0.388
AST, mean±SD (U/L)		24.39 ± 10.77	25.16 ± 13.52	0.797	0.395
AST/ALT, mean±SD		1.25 ± 0.41	1.21 ± 0.44	0.005	0.137
GGT, mean±SD (U/L)		30.43 ± 30.59	41.91 ± 62.50	9.786	< 0.001
CREA, mean±SD (μmol/L)		58.28 ± 14.71	63.87 ± 44.96	5.157	0.016
GLU, mean±SD (mmol/L)		5.19 ± 1.65	5.49 ± 1.36	1.116	0.006
TCH, mean±SD (mmol/L)		5.53 ± 1.10	5.92 ± 1.41	7.260	< 0.001
TG, mean±SD (mmol/L)		1.53 ± 0.89	1.89 ± 1.52	2.968	< 0.001
LDL, mean±SD (mmol/L)		3.28 ± 0.85	3.57 ± 1.03	9.859	< 0.001
HDL, mean±SD (mmol/L)		1.61 ± 0.38	1.61 ± 0.43	1.964	0.848
ApoA1, mean±SD (g/L)		1.40 ± 0.32	1.47 ± 0.41	16.020	0.003
ApoB, mean±SD (g/L)		0.87 ± 0.22	0.98 ± 0.28	16.122	< 0.001
NEFA, mean±SD (mmol/L)		0.47 ± 0.27	0.50 ± 0.29	4.009	0.082
Lp(a), mean±SD (mmol/L)		150.66 ± 161.53	162.86 ± 158.78	1.050	0.300
ACE, mean±SD (U/L)		43.01 ± 18.69	41.94 ± 19.14	0.000	0.441
HCY, mean±SD (μmol/L)		13.27 ± 8.69	14.55 ± 10.29	2.858	0.070
UA, mean±SD (μmol/L)		313.95 ± 96.65	339.13 ± 105.57	0.082	< 0.001
P, mean±SD (mmol/L)		1.10 ± 0.22	1.09 ± 0.23	0.124	0.591
ZN, mean±SD (μmol/L)		10.20 ± 2.10	10.44 ± 2.25	1.164	0.149
MG, mean±SD (mmol/L)		1.05 ± 0.18	1.07 ± 0.18	0.947	0.108
CA, mean±SD (mmol/L)		2.42 ± 0.35	2.46 ± 0.35	0.910	0.055
CU, mean±SD (μmol/L)		18.28 ± 3.58	19.13 ± 3.48	0.312	0.001
FE, mean±SD (μmol/L)		19.06 ± 7.34	19.75 ± 7.77	1.028	0.211
LDH, mean±SD (U/L)		183.14 ± 43.85	190.75 ± 45.84	0.002	0.021
Regular consumption of certain foodstuffs					
Rice, N (%)	No	34 (10.33)	50 (11.68)	0.343	0.558
	Yes	295 (89.67)	378 (88.32)		
Wheat, N (%)	No	25 (7.60)	38 (8.88)	0.399	0.527
	Yes	304 (92.40)	390 (91.12)		
Corn, N (%)	No	250 (75.99)	325 (75.93)	0.000	0.986
	Yes	79 (24.01)	103 (24.07)		
Meat, N (%)	No	218 (66.26)	287 (67.06)	0.053	0.818
	Yes	111 (33.74)	141 (32.94)		
Poultry, N (%)	No	319 (96.96)	418 (97.66)	0.357	0.550
	Yes	10 (3.04)	10 (2.34)		
Fish, N (%)	No	314 (95.44)	417 (97.43)	2.219	0.136
	Yes	14 (4.26)	11 (2.57)		
Egg, N (%)	No	196 (59.57)	268 (62.62)	0.726	0.394
	Yes	133 (40.43)	160 (37.38)		
Vegetable, N (%)	No	51 (15.50)	48 (11.21)	3.007	0.083
	Yes	278 (84.50)	380 (88.79)		
Soya, N (%)	No	257 (78.12)	338 (78.97)	0.081	0.776
	Yes	72 (21.88)	90 (21.03)		
Pickled, N (%)	No	130 (39.51)	166 (38.79)	0.041	0.839
	Yes	199 (60.49)	262 (61.21)		
Fruit, N (%)	No	167 (50.76)	233 (54.44)	1.011	0.315
	Yes	162 (49.24)	195 (45.56)		
Dairy, N (%)	No	301 (91.49)	400 (93.46)	1.052	0.305
	Yes	28 (8.51)	28 (6.54)		
Spicy, N (%)	No	245 (74.47)	312 (72.90)	0.236	0.627
	Yes	84 (25.53)	116 (27.10)		
Supply, N (%)	No	277 (84.19)	343 (80.14)	2.063	0.151
	Yes	52 (15.81)	85 (19.86)		
Average monthly consumption of edible oil, mean±SD (500g/month)		2.57 ± 1.58	2.43 ± 1.54	0.787	0.237
Monthly average salt consumption, mean±SD (g/month)		161.71 ± 89.05	167.89 ± 81.97	5.304	0.328

K-S test the normality of continuous variables, and all continuous variables meet the normal distribution, continuous variable showed in mean ± SD using t-test; categorical variable showed in N (%), using Chi-square test.

**Table 2:** Risk factors for essential hypertension in all subjects

Characteristics	Control (n = 329) Mean ± SD	Case (n = 428) Mean ± SD	*OR*	*95%CI*	*p*
Age (year)	52.98 ± 8.73	57.90 ± 7.64	1.076	(1.056, 1.097)	< 0.001
Waist (cm)	84.64 ± 8.45	88.85 ± 9.36	1.055	(1.037, 1.073)	< 0.001
BMI (kg/m^2^)	24.26 ± 2.89	25.62 ± 3.39	1.149	(1.094, 1.206)	< 0.001
ALP (U/L)	79.99 ± 24.44	89.54 ± 29.44	1.013	(1.008, 1.019)	< 0.001
CREA (μmol/L)	58.28 ± 14.71	63.87 ± 44.96	1.009	(1.001, 1.018)	0.032
GLU (mmol/L)	5.19 ± 1.65	5.49 ± 1.36	1.202	(1.051, 1.374)	0.007
TG (mmol/L)	1.53 ± 0.89	1.89 ± 1.52	1.454	(1.226, 1.723)	< 0.001
LDL (mmol/L)	3.28 ± 0.85	3.57 ± 1.03	1.401	(1.194, 1.644)	< 0.001
ApoA1 (g/L)	1.40 ± 0.32	1.47 ± 0.41	1.789	(1.198, 2.672)	0.004
ApoB (g/L)	0.87 ± 0.22	0.98 ± 0.28	5.408	(2.936, 9.962)	< 0.001
UA (μmol/L)	313.95 ± 96.65	339.13 ± 105.57	1.003	(1.001, 1.004)	< 0.001
CA (mmol/L)	2.42 ± 0.35	2.46 ± 0.35	1.498	(0.987, 2.273)	0.058
CU (μmol/L)	18.28 ± 3.58	19.13 ± 3.48	1.072	(1.028, 1.118)	0.001

**Table 3:** Distribution and risk assessment of EH for each genotype (*AGT, LPR6, PPARG*)

SNP	loci	Genetic model		Control N (%)	Case N (%)	*χ^2^*	*p_1_*	*OR (95CI)*	*p_2_*	*p_adj_*
*AGT*	*rs5046*	codominant	GG	236 (71.7)	311 (72.7)	3.98	0.14			
			GA	89 (27.1)	103 (24.0)			1.17 (0.62,2.18)	0.63	0.88
			AA	4 (1.2)	14 (3.3)			5.49 (1.08,27.97)	**0.04**	0.88
		dominant	GG	236 (71.7)	311 (72.7)	0.78	0.81			
			GA+AA	93 (28.3)	117 (26.3)			1.01 (0.70,10.41)	0.98	0.96
		recessive	GG+GA	325 (98.8)	414 (96.7)	3.39	0.09			
			AA	4 (1.2)	14 (3.3)			3.82 (1.08,13.50)	0.07	0.71
		overdominant	GG+AA	240 (72.9)	325 (76.0)	0.88	0.36			
			GA	89 (27.1)	103 (24.0)			0.88 (0.61,1.26)	0.48	0.74
		additive	GG	236 (98.3)	311 (96.1)	3.12	0.09			
			AA	4 (1.7)	14 (3.9)			3.47 (0.98,12.36)	0.06	0.75
*LRP6*	*rs12823243*	codominant	AA	177 (53.8)	249 (58.2)	3.05	0.23			
			TA	122 (37.1)	153 (35.7)			0.36 (0.11,1.17)	0.09	0.88
			TT	30 (9.1)	26 (6.1)			0.01 (0.01,0.30)	**0.01**	0.88
		dominant	AA	177 (53.8)	249 (58.2)	1.45	0.24			
			TA+TT	152 (46.2)	179 (41.8)			0.78 (0.57,1.08)	0.13	0.74
		recessive	AA+TA	299 (90.9)	402 (93.9)	2.52	0.12			
			TT	30 (9.1)	26 (6.1)			0.54 (0.29,0.99)	**0.04**	0.71
		overdominant	AA+TT	207 (62.9)	275 (64.3)	0.14	0.76			
			TA	122 (37.1)	153 (35.7)			0.92 (0.66,1.28)	0.63	0.86
		additive	AA	177 (85.5)	249 (90.5)	2.92				
			TT	30 (14.5)	26 (9.5)			0.53 (0.28,1.00)	0.05	0.63
*PPARG*	*rs3856806*	codominant	CC	192 (58.4)	282 (65.9)	5.38	0.07			
			CT	118 (35.8)	131 (30.6)			0.79 (0.52,1.20)	0.27	0.88
			TT	19 (5.8)	15 (3.5)			0.45 (0.10,2.09)	0.13	0.88
		dominant	CC	192 (58.4)	282 (65.9)	4.51	0.03			
			CT+TT	137 (41.6)	146 (34.1)			0.70 (0.51,0.97)	**0.03**	0.74
		recessive	CC+CT	310 (94.2)	413 (96.5)	2.24	0.16			
			TT	19 (5.8)	15 (3.5)			0.58 (0.27,1.23)	0.16	0.82
		overdominant	CC+TT	211 (64.2)	297 (69.4)	2.33	0.14			
			CT	118 (35.8)	131 (30.6)			0.77 (0.55,1.07)	0.12	0.64
		additive	CC	192 (99.1)	282 (99.5)	3.09	0.10			
			TT	19 (0.9)	15 (0.5)			0.52 (0.24,1.15)	0.11	0.63

Chi-square test was used to analyze the distribution of genotypes between case and control(*p_1_*), multifactorial logistic regression (OR, *p_2_*) was used to analyze the influence of genotypes on disease, *p_adj_* was adjusted by FDR-BH correction.

**Table 4:** The result of differences in *ACE2* genotype distribution divided by sex

	ACE2	Genetic model		control	case					
	SNP		genotype	N (%)	N (%)	χ2	p1	OR (95CI)	P2	padj
male	*rs1978124*	codominant	CC	134 (99.3)	202(98.5)	0.71	1			
			TC	0 (0.0)	1 (0.5)			-	-	-
			TT	1 (0.7)	2 (1.0)			1.45 (0.11,18.55)	0.77	0.99
		dominant	CC	134 (99.3)	202 (98.5)	0.37	0.65			
			TC+TT	1 (0.7)	3 (1.5)			1.91 (0.18,20.91)	0.60	0.99
		recessive	CC+TC	134 (99.3)	203 (99.0)	0.05	1			
			TT	1 (0.7)	2 (1.0)			1.45 (0.11,18.51)	0.78	0.99
		overdominant	CC+TT	135 (100.0)	204 (99.5)	0.66	1			
			TC	0 (0.0)	1 (0.5)			-	-	-
		additive	CC	134 (99.3)	202 (99.0)	0.05	1			
			TT	1 (0.7)	2 (1.0)			1.07 (0.97,1.17)	0.16	0.41
	***rs2285666***	codominant	TT	78 (57.8)	101 (49.3)	3.65	0.17			
			CT	11 (8.1)	13 (6.3)			1.04 (0.41,2.63)	0.94	0.99
			CC	46 (34.1)	91 (47.4)			1.70 (1.02,2.83)	**0.04**	0.12
		dominant	TT	78 (57.8)	101(49.3)	2.36	0.15			
			CT+CC	57 (42.2)	104 (50.7)			1.69 (1.05,2.71)	**0.03**	0.12
		recessive	TT+CT	89 (65.9)	114 (55.6)	3.60	0.07			
			CC	46 (34.1)	91 (44.4)			1.79 (1.11,2.90)	**0.02**	0.12
		overdominant	TT+CC	124 (91.9)	192 (93.7)	0.41	0.67			
			CT	11 (8.1)	13 (6.3)			0.86 (0.35,2.08)	0.73	0.99
		additive	TT	78 (62.9)	101 (52.6)	3.25	0.08			
			CC	46 (37.1)	91 (47.4)			1.07 (0.97,1.18)	0.18	0.41
female	***rs2285666***	codominant	TT	74 (38.1)	66 (29.6)	4.53	0.10			
			CT	80 (41.2)	114 (51.1)			1.76 (1.02,3.03)	**0.04**	0.12
			CC	40 (20.7)	43 (19.3)			1.36 (0.69,2.70)	0.38	0.76
		dominant	TT	74 (38.1)	66 (29.6)	3.40	0.07			
			CT+CC	120 (61.9)	157 (70.4)			1.69 (1.02,2.80)	**0.04**	0.12
		recessive	TT+CT	154 (79.3)	180 (80.7)	0.12	0.81			
			CC	40 (20.7)	43 (19.3)			0.96 (0.54,1.70)	0.88	0.99
		overdominant	TT+CC	114 (58.8)	109 (48.9)	4.07	**0.04**			
			CT	80 (41.2)	114 (51.1)			0.96 (0.54,1.70)	0.88	0.99
		additive	TT	74 (64.9)	66 (60.6)	0.45	0.58			
			CC	40 (35.1)	43 (39.4)			1.00 (0.90,1.11)	0.97	0.99

Chi-square test was used to compare the distribution difference of alleles between the two groups, and N(%) was used to describe the data distribution. The p-value of Chi-square test was *p_1_*, that of multivariate logistic regression was *p_2_*. *p_adj_* was adjusted by FDR-BH correction.

**Table 5:** The Results of haplotype difference analyses

Block	loci	Type	Control (%)	Case (%)	*OR (95%CI)*	*p*
*AGT*	*rs699* *rs2004776* *rs2493134* *rs3789679*	GTCA	49.5	38.3	-	0.64
		GCCG	14.3	20.9	1.66 (0.67,4.12)	0.28
		ACTG	19.8	17.4	1.14(0.48,2.69)	0.77
		GTCG	16.5	23.5	1.56 (0.70,3.59)	0.32
*ACE*	*rs4316* *rs4343*	TA	78.4	74.4	-	-
		CG	21.6	25.6	1.19 (0.68,2.10)	0.49
*AGTR1*	*rs275646* *rs2933249* *rs1492100*	GAT	94.9	94.5	-	0.94
		GAA	1.1	0.8	1.69 (0.21,13.77)	0.62
		AGT	3.4	4.3	1.09 (0.35,3.37)	0.88
		AAT	0.6	0.4	1.71 (0.10,28.72)	0.71
*AGTR1*	*rs5182* *rs5186* *rs275646*	TAC	98.4	94.4	-	0.42
		CAT	1.0	2.4	2.58 (0.48,13.87)	0.27
		CAC	0.0	2.8	-	1
		CCC	0.5	0.4	0.16 (0.01,2.80)	0.21
*CYP11B2*	*rs3802228* *rs6433* *rs1799998*	ATA	65.0	67.5	-	0.95
		GTG	25.8	24.0	0.93 (0.47,1.83)	0.82
		ACA	8.3	7.8	0.74 (0.24,2.29)	0.60
		GTA	0.8	7.8	1.62 (0.05,54.08)	0.79
*LRP6*	*rs11054731* *rs10743980*	GC	84.5	87.5	-	0.46
		AT	14.4	11.6	0.71 (0.37,1.37)	0.31
		AC	1.1	0.9	0.46 (0.06,3.47)	0.45
*PPARG*	*rs2972164* *rs9817428* *rs12631819*	CAT	42.2	38.0	-	0.51
		CCG	39.2	45.7	1.52 (0.81,3.12)	0.18
		CAG	15.7	13.2	0.94 (0.37,2.37)	0.89
		TCG	2.9	3.1	1.03 (0.15,6.67)	1

**Table 6:** Gene scoring results based on the regression model

Term	Median	*OR*	*95%CI*	*p*
Genetic score for whole genes	-0.225	1.229	1.110,1.362	< 0.01
Two-gene genetic score	1	0.902	0.732,1.111	0.332

**Table 7:** Results of log-rank test

Term	*χ^2^*	*OR (95%CI)*	*p*
Log Rank (Mantel-Cox)	20.283	2.150 (1.998,2.377)	< 0.01

**Table 8:** The stratification analysis between genetic score and age

		Control	Case	*χ2*	*OR (95%CI)*	*p*
Gene score ≤ -0.225	*Age < 60	23	224	0.817	1.0	0.366
	*Age ≥ 60	5	77		0.632 (0.232,1.721)	
						
Gene score > -0.225	*Age < 60	20	214	5.582	1.0	0.018
	*Age ≥ 60	31	163		2.035 (1.119,3.700)	
