# Supplementary material for: The RAAS system SNPs polymorphism is associated with essential hypertension risk in rural areas in northern China
Source: Int J Med Sci. 2024 Oct 14;21(14):2694–704. doi: 10.7150/ijms.98724 (PMC11539379; doi:10.7150/ijms.98724)
Supplement: Supplementary file 1 — Supplementary information and tables. [file ijmsv21p2694s1.pdf]

Exclusion criteria:

① Case group: secondary hypertension, diabetes, thyroid diseases, severe liver and kidney diseases, malignant tumors, cardiovascular disease (CVD) such as heart failure, atrial fibrillation, myocardial infarction or stroke, and subjects who have recently taken birth control pills or other drugs that may affect blood pressure.

② Control group: people who have a history of hypertension and have used antihypertensive drugs for CVD such as heart failure, atrial fibrillation, myocardial infarction, severe arrhythmia or stroke, diabetes, thyroid diseases, severe liver and kidney diseases, severe varicose veins of the lower extremities, and malignant tumors, and subjects who have recently taken birth control pills or other drugs that may affect blood pressure.

Table S1. Criterion for the factors and acronyms for biochemical measures

Characteristics	criterion
Sociodemographic factors	
Marital status	Unmarital or marital
Nine-year compulsory education	Over nine-year compulsory education or not
Lifestyle behaviors	
Current smoking	Never smoking and quitting smoking are defined as non-smoking; smoking is defined as current smoking
Passive smoking	passively inhaled cigarettes at home or in work environment
Harmful drinking	pure alcohol intake= alcohol content multiplied by alcohol consumption(ml), male≥61g/day or female≥41g/day is defined as harmful drinking
Physical exertion	at least 150 min of moderate or 75 min of vigorous activity per week is defined as physical exertion
Exercise regularly	over three times per week is defined as exercise regularly
Regular consumption of certain foodstuffs	over three times per week is defined as frequent consumption
Acronyms of Biochemical measures	
ALP	Alkaline phosphatase
ALT	Alanine aminotransferase
AST	Aspartate aminotransferase
AST/ALT	Aspartate aminotransferase/ Alanine aminotransferase
GGT	Gamma-glutamyltransferase
CREA	Creatinine
GLU	Glucose
TCH	Total cholesterol
TG	Triglyceride
LDL	Low density lipoprotein cholesterol
HDL	High density lipoprotein cholesterol
ApoA1	Apolipoprotein A-I
ApoB	Apolipoprotein B
NEFA	Nonestesterified fatty acid
Lp(a)	Lipoprotein(a)
ACE	Angiotensin converting enzyme
HCY	Homocysteine
UA	Uric acid
P	Phosphorus
ZN	Zinc
MG	Magnesium
CA	Calcium
CU	Copper
FE	Iron
LDH	Serum lactate dehydrogenase

Table S2. Demographic characteristics and serum index determination for female subjects

Characteristics		Control	Case	$T/Z/\chi^2$	p
BP					
SBP, mean ± SD (mmHg)		122.45 ± 10.17	154.47 ± 16.82	27.805	0.000
DBP, mean ± SD (mmHg)		78.98 ± 7.19	93.87 ± 10.68	13.689	0.000
Sociodemographic factors					
Age, mean ± SD (year)		51.50±8.11	57.28±7.51	0.060	0.000
Marital status, N (%)	Unmarried	13(6.70)	20(8.97)	0.732	0.392
	Married	181(93.30)	203(91.03)		
Nine-year compulsory education, N (%)	Uncompleted	178(91.75)	204(91.48)	0.010	0.920
	Completed	16(8.25)	19(8.52)		
Lifestyle behaviors					
Current smoking, N (%)	No	127(65.46)	150(67.26)	0.151	0.698

	Yes	67(34.54)	73(32.74)		
Passive smoking, N (%)	No	105(54.12)	115(51.57)	0.272	0.602
	Yes	89(45.88)	108(48.43)		
Harmful drinking, N (%)	No	191(98.45)	220(98.65)	0.030	0.863
	Yes	3(1.55)	3(1.35)		
Physical exertion, N (%)	Light	135(69.59)	154(69.06)	2.817	0.244
	Medium	9(4.64)	19(8.52)		
	Heavy	50(25.77)	50(22.42)		
Exercise regularly, N (%)	No	144(74.23)	173(77.58)	0.639	0.424
	Yes	50(25.77)	50(22.42)		
Adiposity measures					
Waist, mean ± SD (cm)		82.38±7.72	87.62±9.87	4.742	0.000
Hip, mean ± SD (cm)		95.19±7.42	98.76±8.49	2.721	0.000
WHR, mean ±SD		0.87±0.09	0.89±0.11	0.975	0.027
BMI, median(IQR) (kg/m²)		23.62（22.09,26.02）	25.73(23.46,27.89)	-5.521	＜0.001
Medical history					
Family history of hypertension, N(%)	No	154(79.38)	177(79.37)	0.000	0.998
	Yes	40(20.62)	46(20.63)		
Family history of diabetes, N (%)	No	183(94.33)	211(94.62)	0.017	0.897
	Yes	11(5.67)	12(5.38)		
Family history of Cardiovascular disease, N (%)	No	179(92.27)	209(93.72)	0.339	0.560
	Yes	15(7.73)	14(6.28)		
Clinical and biochemical measures					
ALP, mean ± SD (U/L)		81.21±27.25	93.67±30.78	0.716	0.000
ALT, mean ± SD (U/L)		19.82±14.92	20.83±10.95	0.384	0.424
AST, mean ± SD (U/L)		22.94±10.41	22.98±6.87	0.231	0.964
AST/ALT, mean ± SD		1.31±0.40	1.25±0.47	0.362	0.205
GGT, mean ± SD (U/L)		23.62±21.40	26.79±15.90	0.388	0.085
CREA, mean ±SD (μmol/L)		52.02±10.30	58.52±57.01	4.543	0.096
GLU, mean ±SD (mmol/L)		5.01±0.67	5.37±1.24	11.376	0.000
TCH, mean ±SD (mmol/L)		5.43±0.92	6.01±1.51	13.428	0.000
TG, mean ±SD (mmol/L)		1.50±0.79	1.95±1.01	8.984	0.000
LDL, mean ±SD (mmol/L)		3.19±0.71	3.66±1.11	21.538	0.000
HDL, mean ± SD (mmol/L)		1.60±0.36	1.58±0.40	1.256	0.700
ApoA1, mean ± SD (g/L)		1.37±0.27	1.47±0.39	17.251	0.003
ApoB, mean ± SD (g/L)		0.84±0.19	1.00±0.29	23.724	0.000
NEFA, median (IQR) (mmol/L		0.49（0.31,0.66）	0.53(0.31,0.71)	-1.525	0.127
Lp(a), mean ± SD (mmol/L)		155.52±173.32	172.77±169.67	0.478	0.307
ACE, mean ± SD (U/L)		43.55±18.29	42.34±18.91	0.015	0.511
HCY, mean ± SD (μmol/L)		11.29±6.71	12.05±5.91	0.038	0.216
UA, mean ± SD (μmol/L)		277.46±75.42	298.89±73.99	0.001	0.004
P, mean ± SD (mmol/L)		1.14±0.20	1.16±0.22	0.582	0.351
ZN, mean ± SD (μmol/L)		10.30±2.01	10.43±2.08	0.320	0.514
MG, mean ± SD (mmol/L)		1.04±0.17	1.09±0.18	1.876	0.005
CA, mean ± SD (mmol/L)		2.40±0.31	2.48±0.34	4.956	0.012
CU, mean ± SD (μmol/L)		18.97±3.67	20.08±3.48	0.047	0.002
FE, mean ± SD (μmol/L)		17.11±6.25	17.22±6.38	0.166	0.857
LDH, mean ± SD (U/L)		186.98±43.35	199.17±49.28	0.773	0.008
Regular consumption of certain foodstuffs					
Rice, N(%)	No	17(8.76)	25(11.21)	0.686	0.407
	Yes	177(91.24)	198(88.79)		
Wheat, N(%)	No	14(7.22)	20(8.97)	0.425	0.514
	Yes	180(92.78)	203(91.03)		
Corn, N(%)	No	149(76.80)	164(73.54)	0.590	0.443
	Yes	45(23.20)	59(26.46)		
Meat, N(%)	No	146(75.26)	168(75.34)	0.000	0.985
	Yes	48(24.74)	55(24.66)		
Poultry, N(%)	No	191(98.45)	222(99.55)	1.316	0.251
	Yes	3(1.55)	1(0.45)		
Fish, N(%)	No	189(97.42)	216(96.86)	0.117	0.732
	Yes	5(2.58)	7(3.14)		
Egg, N(%)	No	119(61.34)	150(67.26)	1.590	0.207
	Yes	75(38.66)	73(32.74)		
Vegetable, N(%)	No	35(18.04)	21(9.42)	6.637	0.010
	Yes	159(81.96)	202(90.58)		
Soya, N(%)	No	158(81.44)	181(81.17)	0.005	0.942
	Yes	36(18.56)	42(18.83)		
Pickled, N(%)	No	72(37.11)	86(38.57)	0.093	0.761
	Yes	122(62.89)	137(61.43)		
Fruit, N(%)	No	100(51.55)	116(52.02)	0.009	0.923
	Yes	94(48.45)	107(47.98)		
Dairy, N(%)	No	179(92.27)	212(95.07)	1.390	0.238
	Yes	15(7.73)	11(4.93)		
Spicy, N(%)	No	149(76.80)	165(73.99)	0.441	0.506
	Yes	45(23.20)	58(26.01)		

Supply, N(%)	No<br>Yes	165(85.05)<br>29(14.95)	177(79.37)<br>46(20.63)	2.269	0.132
Average monthly consumption of edible oil, mean ± SD (500g/month)		2.53±1.53	2.48±1.59	0.092	0.761
Monthly average salt consumption, mean ± SD (g/month)		154.63±85.39	168.94±80.84	0.967	0.080

K-S test the normality of continuous variables, the normal distribution showed in mean ± SD, the abnormal distribution showed in median (IQR); continuous variable showed in mean ± SD or median (IQR), using t-test or Mann-Whitney U; categorical variable showed in N(%),using chi-square test.

Table S3. Demographic characteristics and serum index determination for male subjects

Characteristics		Control (n = 135)	Case (n = 205)	T/χ^2	*p*
BP					
SBP, mean ± SD, mmHg		122.71±9.56	152.38±18.31	32.949	0.000
DBP, mean ± SD, mmHg		81.51±5.49	96.98±11.12	24.784	0.000
Sociodemographic factors					
Age, mean ± SD, y		55.10±9.18	58.59±7.74	5.801	0.000
Marital status, N(%)	Unmarried<br>Married	9(6.67)<br>126(93.33)	19(9.27)<br>186(90.73)	0.729	0.393
Nine-year compulsory education, N (%)	Uncompleted<br>Completed	112(82.96)<br>23(17.04)	171(83.41)<br>34(16.59)	0.012	0.913
Lifestyle behaviors					
Current smoking, N(%)	No<br>Yes	60(44.44)<br>75(55.56)	113(55.12)<br>92(44.88)	3.713	0.054
Passive smoking, N(%)	No<br>Yes	90(66.67)<br>45(33.33)	129(62.93)<br>76(37.07)	0.497	0.481
Harmful drinking, N(%)	No<br>Yes	73(54.07)<br>62(45.93)	112(54.63)<br>93(45.37)	0.010	0.919
Physical exertion, N(%)	Light<br>Medium<br>Heavy	75(55.56)<br>9(6.67)<br>51(37.78)	109(53.17)<br>15(7.32)<br>81(39.51)	0.197	0.906
Exercise regularly, N(%)	No<br>Yes	84(62.22)<br>51(37.78)	124(60.49)<br>81(39.51)	0.103	0.748
Adiposity measures					
Waist, mean ± SD, cm		87.90±8.41	90.19±8.60	0.002	0.016
Hip, mean ± SD, cm		100.17±7.49	101.29±6.96	0.032	0.159
WHR, mean ± SD		0.88±0.12	0.89±0.09	0.580	0.386
BMI, mean ± SD, kg/m²		24.85±2.94	25.44±3.00	0.078	0.075
Medical history					
Family history of hypertension, N(%)	No<br>Yes	113(83.70)<br>22(16.30)	169(82.44)<br>36(17.56)	0.092	0.762
Family history of diabetes, N(%)	No<br>Yes	128(94.81)<br>7(5.19)	202(98.54)<br>3(1.46)	3.950	0.047
Family history of cardiovascular disease, N(%)	No<br>Yes	130(96.30)<br>5(3.70)	194(94.63)<br>11(5.37)	0.501	0.479
Clinical and biochemical measures					
ALP, mean ± SD, U/L		78.24±19.69	85.05±27.29	9.556	0.008
ALT, mean ± SD, U/L		25.86±17.53	25.85±15.53	0.044	0.995
AST, mean ± SD, U/L		26.46±10.97	27.53±17.90	1.930	0.535
AST/ALT, mean ± SD		1.17±0.40	1.16±0.39	0.315	0.685
GGT, mean ± SD, U/L		40.22±38.30	58.36±85.91	9.969	0.008
CREA, mean ± SD (μmol/L)		67.28±15.44	69.69±25.08	1.233	0.319
GLU, mean ± SD (mmol/L)		5.44±2.43	5.61±1.48	0.803	0.427
TCH, mean ± SD (mmol/L)		5.67±1.30	5.82±1.31	0.038	0.294
TG, mean ± SD (mmol/L)		1.56±1.01	1.83±1.92	0.070	0.139
LDL, mean ± SD (mmol/L)		3.40±1.00	3.48±0.93	0.214	0.464
HDL, mean ± SD (mmol/L)		1.62±0.40	1.64±0.46	0.484	0.608
ApoA1, mean ± SD, g/L		1.43±0.38	1.48±0.42	1.931	0.279
ApoB, mean ± SD, g/L		0.91±0.26	0.95±0.27	0.225	0.199
NEFA, mean ± SD (mmol/L)		0.43±0.26	0.47±0.29	1.321	0.222
Lp(a), mean ± SD (mmol/L)		143.72±143.36	151.92±145.45	0.719	0.610
ACE, mean ± SD, U/L		42.25±19.29	41.50±19.43	0.007	0.730
HCY, mean ± SD (μmol/L)		16.11±10.31	17.26±13.01	2.856	0.388
UA, mean ± SD (μmol/L)		366.39±99.87	382.90±116.95	0.455	0.179
P, mean ± SD (mmol/L)		1.04±0.23	1.01±0.21	0.335	0.307
ZN, mean ± SD (μmol/L)		10.07±2.23	10.44±2.43	0.649	0.153
MG, mean ± SD (mmol/L)		1.08±0.19	1.06±0.18	0.052	0.457
CA, mean ± SD (mmol/L)		2.44±0.41	2.45±0.36	1.099	0.796
CU, mean ± SD (μmol/L)		17.29±3.20	18.11±3.18	0.001	0.021
FE, mean ± SD (μmol/L)		21.85±7.88	22.50±8.22	1.301	0.467
LDH, mean ± SD, U/L		177.63±44.13	181.58±39.92	3.202	0.392
Regular consumption of certain foodstuffs					
Rice, N(%)	No	17(12.59)	25(12.20)	0.012	0.913

	Yes	118(87.41)	180(87.80)		
Wheat, N(%)	No	11(8.15)	18(8.78)	0.042	0.838
	Yes	124(91.85)	187(91.22)		
Corn, N(%)	No	101(74.81)	161(78.54)	0.638	0.425
	Yes	34(25.19)	44(21.46)		
Meat, N(%)	No	72(53.33)	119(58.05)	0.735	0.391
	Yes	63(46.67)	86(41.95)		
Poultry, N(%)	No	128(94.81)	196(95.61)	0.115	0.735
	Yes	7(5.19)	9(4.39)		
Fish, N(%)	No	125(92.59)	201(98.05)	6.138	0.013
	Yes	10(7.41)	4(1.95)		
Egg, N(%)	No	77(57.04)	118(57.56)	0.009	0.924
	Yes	58(42.96)	87(42.44)		
Vegetable, N(%)	No	16(11.85)	27(13.17)	0.128	0.720
	Yes	119(88.15)	178(86.83)		
Soya, N(%)	No	99(73.33)	157(76.59)	0.463	0.496
	Yes	36(26.67)	48(23.41)		
Pickled, N(%)	No	58(42.96)	80(39.02)	0.524	0.469
	Yes	77(57.04)	125(60.98)		
Fruit, N(%)	No	67(49.63)	117(57.07)	1.816	0.178
	Yes	68(50.37)	88(42.93)		
Dairy, N(%)	No	122(90.37)	188(91.71)	0.181	0.671
	Yes	13(9.63)	17(8.29)		
Spicy, N(%)	No	96(71.11)	147(71.71)	0.014	0.905
	Yes	39(28.89)	58(28.29)		
Supply, N(%)	No	112(82.96)	166(80.98)	0.216	0.642
	Yes	23(17.04)	39(19.02)		
Average monthly consumption of edible oil, mean ± SD, (500g/month)		2.62±1.65	2.37±1.47	2.724	0.156
Monthly average salt consumption, mean ± SD, (g/month)		171.87±93.44	166.74±83.37	4.950	0.606

K-S test the normality of continuous variables, and all continuous variables meet the normal distribution，continuous variable showed in mean ± SD, using t-test；categorical variable showed in N (%), using a chi-square test.

Table S4. Risk factors for essential hypertension in Collinearity diagnosis

Characteristics	Tolerance	Variance Inflation Factor (VIF)	Condition Index (CI)
Age(year)	0.837	1.195	6.243
Waist(cm)	0.465	2.149	7.537
BMI (kg/m^2)	0.485	2.062	11.211
ALP(U/L)	0.675	1.482	11.968
CREA(μmol/L)	0.800	1.250	13.473
GLU (mmol/L)	0.895	1.117	15.684
TG (mmol/L)	0.845	1.183	16.668
LDL (mmol/L)	0.234	4.280	21.227
ApoA1(g/L)	0.567	1.764	26.200
ApoB (g/L)	0.198	5.058	33.147
UA (μmol/L)	0.623	1.606	42.801
CA (mmol/L)	0.442	2.264	56.852
CU(μmol/L)	0.684	1.462	65.955

Table S5. Risk factors for essential hypertension in female subjects

Characteristics	Control (n=194)<br>Mean ±SD<br>Median (IQR)	Case (n=223)<br>Mean ±SD<br>Median (IQR)	*OR*	*95%CI*	*p*
Age（year）	51.50±8.11	57.28±7.51	1.098	[1.069,1.128]	＜0.001
Waist (cm)	82.38±7.72	87.62±9.87	1.070	[1.045,1.096]	＜0.001
BMI (kg/m^2)	23.62（22.09,26.02）	25.73(23.46,27.89)	1.204	[1.128,1.286]	＜0.001
ALP (U/L)	81.21±27.25	93.67±30.78	1.015	[1.008,1.022]	＜0.001
CREA(μmol/L)	52.02±10.30	58.52±57.01	1.009	[0.995,1.024]	0.218
GLU (mmol/L)	5.01±0.67	5.37±1.24	1.577	[1.216,2.045]	＜0.001
TG (mmol/L)	1.50±0.79	1.95±1.01	1.812	[1.407,2.334]	＜0.001
LDL (mmol/L)	3.19±0.71	3.66±1.11	1.766	[1.398,2.230]	＜0.001
ApoA1(g/L)	1.37±0.27	1.47±0.39	2.399	[1.322,4.356]	0.004
ApoB (g/L)	0.84±0.19	1.00±0.29	16.702	[6.593,42.314]	＜0.001
UA (μmol/L)	277.46±75.42	298.89±73.99	1.004	[1.001,1.007]	0.004
CA (mmol/L)	2.40±0.31	2.48±0.34	2.188	[1.165,4.109]	0.015
CU (μmol/L)	18.97±3.67	20.08±3.48	1.092	[1.033,1.155]	0.002

Table S6. Risk factors for essential hypertension in male subjects

Characteristics	Control (n=135)<br>Mean ± SD	Case (n=205)<br>Mean ± SD	*OR*	*95%CI*	*p*
Age (year)	55.10±9.18	58.59±7.74	1.051	[1.023,1.080]	＜0.001
Waist (cm)	87.90±8.41	90.19±8.60	1.032	[1.005,1.060]	0.018
BMI (kg/m^2)	24.85±2.94	25.44±3.00	1.070	[0.993,1.152]	0.076
ALP (U/L)	78.24±19.69	85.05±27.29	1.012	[1.002,1.021]	0.014
CREA (μmol/L)	67.28±15.44	69.69±25.08	1.006	[0.994,1.017]	0.326
GLU (mmol/L)	5.44±2.43	5.61±1.48	1.053	[0.925,1.198]	0.434
TG (mmol/L)	1.56±1.01	1.83±1.92	1.187	[0.946,1.489]	0.138
LDL (mmol/L)	3.40±1.00	3.48±0.93	1.090	[0.867,1.370]	0.463
ApoA1 (g/L)	1.43±0.38	1.48±0.42	1.352	[0.784,2.332]	0.279
ApoB (g/L)	0.91±0.26	0.95±0.27	1.740	[0.747,4.053]	0.199
UA (μmol/L)	366.39±99.87	382.90±116.95	1.001	[0.999,1.003]	0.180
CA (mmol/L)	2.44±0.41	2.45±0.36	1.078	[0.610,1.907]	0.795
CU (μmol/L)	17.29±3.20	18.11±3.18	1.085	[1.012,1.164]	0.023

Table S7. The SNPs information and the result of Hardy-Weinberg equilibrium test

SNP	Loci	Chr<br>/Posstion	Region	Major/<br>minor<br>allele	genotype(n)<br>11	12	22	χ^2	*p*	MAF(%)
AGT	*rs699*	1/230845794	nonsynon_exon2	G/A	445	274	38	0.252	0.616	23.1
	rs2493134	1/230849359	intron1	C/T	444	275	38	0.301	0.583	23.2
	rs2004776	1/230848702	intron1	T/C	218	389	150	0.987	0.321	45.5
	rs2148582	1/230849799	intron1	G/A	444	275	38	0.301	0.583	23.2
	rs5046	1/230850398	5'-flanking	G/A	547	192	18	0.056	0.813	15.1
	rs3789679	1/230849694	intron1	G/A	349	319	89	1.498	0.221	32.8
ACE	*rs4316*	17/61562309	intron12	T/C	308	352	97	0.052	0.819	36.1
	rs4343	17/61566031	synon_exon16	A/G	305	355	97	0.159	0.690	36.3
	rs4461142	17/61578048	3'-flanking	C/T	238	374	145	0.008	0.929	43.9
AGTR1	*rs5182*	3/148459395	synon_exon3	T/C	411	291	55	0.125	0.724	26.5
	rs1492100	3/148437427	intron2	T/A	435	268	54	2.026	0.155	10.6
	rs5186	3/148459988	3'-UTR_exon3	A/C	686	69	2	0.036	0.849	5.1
	rs275646	3/148463522	3'-flanking	C/T	636	118	3	1.009	0.315	8.3
	rs2933249	3/148416520	intron1	G/A	571	173	13	0.001	0.980	13.1
	rs2638360	3/148428356	intron2	A/G	609	137	11	1.051	0.305	10.5
CYP11B2	*rs6433*	8/143993640	intron8	T/C	529	205	23	0.332	0.546	16.6
	rs3802228	8/143992218	3'-UTR_exon9	A/G	312	362	83	2.106	0.147	34.9
	rs1799998	8/143999600	5'-flanking	A/G	474	249	34	0.032	0.859	31.8
LDLR	*rs688*	19/11227602	synon_exon12	C/T	555	187	15	0.027	0.870	14.3
LRP5	*rs638051*	11/68141414	intron5	A/G	382	311	64	0.004	0.950	29.0
	rs556442	11/68192690	synon_exon15	A/G	397	297	63	0.498	0.480	27.9
LRP6	*rs10743980*	12/12412795	intron1	C/T	436	269	52	1.404	0.236	24.6
	rs11054731	12/12376465	intron2	G/A	351	327	79	0.048	0.826	32.0
	rs2417086	12/12350113	intron3	A/G	435	268	54	2.026	0.155	24.8
	rs7136900	12/12423093	5'-flanking	G/A	627	125	5	0.208	0.648	8.9
	rs12823243	12/12329686	intron7	A/T	426	275	56	1.560	0.212	25.6
PPARG	*rs3856806*	3/12475557	synon_exon7	C/T	474	249	34	0.032	0.859	20.9
	rs1175543	3/12466433	intron6	A/G	245	364	148	0.375	0.540	43.6
	rs2972164	3/12334416	intron1	C/T	621	129	7	0.011	0.917	9.4
	rs13433696	3/12358492	intron1	G/A	329	337	91	0.109	0.742	34.3
	rs9817428	3/12340267	intron1	A/C	234	346	159	0.627	0.429	45.0
	rs12631819	3/12342861	intron1	G/T	305	360	92	0.819	0.365	35.9
ACE2(female)	*rs2285666*	X/15610348	intron4	T/C	140	194	83	1.120	0.290	-

Chi-square test was used to compare the distribution difference of alleles between the two groups, and “n” was used to describe the data distribution.

Table S8. The result of comparison of the allele frequency distribution in case and control group

SNP	Loci	allele	contols n(%)	case n(%)	χ^2	*p*	SNP	loci	allele	contols n(%)	case n(%)	χ^2	*p*
AGT	*rs699*	G	511(77.7)	653(76.3)	0.40	0.53	*CYP11B2*	*rs1799998*	A	438(66.6)	594(69.4)	1.37	0.24
		A	147(22.3)	203(23.7)					G	220(33.4)	262(30.6)		
	rs2493134	C	511(77.7)	652(76.2)	0.45	0.50	*LDLR*	*rs688*	C	568(86.3)	729(85.2)	0.41	0.52
		T	147(22.3)	204(23.8)					T	90(13.7)	127(14.8)		
	rs2004776	T	370(56.2)	455(53.2)	1.42	0.23	*LRP5*	*rs638051*	A	469(71.3)	426(63.0)	10.30	<0.01
		C	288(43.8)	401(46.8)					G	189(28.7)	250(37.0)		
	rs2148582	G	511(77.7)	652(76.2)	0.45	0.50		*rs556442*	A	473(71.9)	618(72.2)	0.02	0.89
		A	147(22.3)	204(23.8)					G	185(28.1)	238(27.8)		
	rs5046	G	561(85.3)	725(84.7)	0.09	0.76	*LRP6*	*rs10743980*	C	488(74.2)	653(76.3)	0.90	0.34
		A	97(14.7)	131(15.3)					T	170(25.8)	203(23.7)		
	rs3789679	G	433(65.8)	584(68.2)	0.99	0.32		*rs11054731*	G	436(66.3)	593(69.3)	1.55	0.21
		A	225(34.2)	272(31.8)					A	222(33.7)	263(30.7)		
ACE	*rs4316*	T	424(64.4)	544(63.6)	0.13	0.72		*rs2417086*	A	485(73.7)	653(76.3)	1.32	0.25
		C	234(35.6)	312(36.4)					G	173(26.3)	203(23.7)		
	rs4343	A	423(64.3)	542(63.3)	0.15	0.70		*rs7136900*	G	605(91.9)	774(90.4)	1.06	0.30
		G	235(35.7)	314(36.7)					A	53(8.1)	82(9.6)		
	rs4461142	C	365(55.5)	485(56.7)	0.21	0.64		*rs12823243*	A	476(72.3)	651(76.1)	2.69	0.10
		T	293(44.5)	371(43.3)					T	182(27.7)	205(23.9)		
AGTR1	*rs5182*	T	475(72.2)	638(74.5)	1.05	0.30	*PPARG*	*rs3856806*	C	502(76.3)	695(81.2)	5.40	0.02
		C	183(27.8)	218(25.5)					T	156(23.7)	161(18.8)		
	rs1492100	T	591(89.8)	763(89.1)	0.18	0.67		*rs1175543*	A	369(56.1)	485(56.7)	0.05	0.82
		A	67(10.2)	93(10.9)					G	289(43.9)	371(43.3)		
	rs5186	A	623(94.7)	818(95.6)	0.63	0.43		*rs2972164*	C	595(90.4)	776(90.7)	0.02	0.88
		C	35(5.3)	38(4.4)					T	63(9.6)	80(9.3)		
	rs275646	C	608(92.4)	781(91.2)	0.66	0.41		*rs13433696*	G	420(63.8)	574(67.1)	1.72	0.19
		T	50(7.6)	75(8.8)					A	238(36.2)	282(32.9)		
	rs2933249	G	576(87.5)	739(86.3)	0.47	0.49		*rs9817428*	A	365(55.5)	467(54.6)	0.13	0.72
		A	82(12.5)	117(13.7)					C	293(44.5)	389(45.4)		
	rs2638360	A	594(90.3)	761(88.9)	0.74	0.38		*rs12631819*	G	415(63.1)	555(64.8)	0.50	0.47
		G	64(9.7)	95(11.1)					T	243(36.9)	301(35.2)		
CYP11B2	*rs6433*	T	549(83.4)	714(83.4)	0.01	0.99	*ACE2*	*rs1978124*	C	655(99.5)	851(99.4)	0/01	0.98
		C	109(16.6)	142(16.6)					T	3(0.5)	5(0.6)		
	rs3802228	A	416(63.2)	570(66.6)	1.86	0.17		*rs22856666*	T	395(60.0)	461(53.9)	5.77	0.01
		G	242(36.8)	286(33.4)					C	263(40.0)	395(46.1)		

Chi-square test was used to compare the distribution difference of alleles between the two groups, and n (%) was used to describe the data distribution.

Table S9. Distribution and risk assessment of EH for each genotype

SNP	loci	genetic model		control n(%)	case n(%)	χ^2	p_1	*OR (95CI)*	p_2	p_{adj}
AGT	*rs699*	codominant	GG	200(60.8)	245(57.2)	1.61	0.44			
			AG	111(33.7)	163(38.1)			1.19(0.86,1.65)	0.30	0.88
			AA	18(5.5)	20(4.7)			0.87(0.41,1.81)	0.70	0.88
		dominant	GG	200(60.8)	245(57.2)	0.97	0.33			
			AG+AA	129(38.2)	183(42.8)			1.09(0.79,1.51)	0.61	0.72
		recessive	GG+AG	311(94.5)	408(95.3)	0.25	0.74			
			AA	18(5.5)	20(4.7)			0.80(0.39,1.67)	0.56	0.96
		overdominant	GG+AA	218(66.3)	265(61.9)	1.52	0.22			
			AG	111(33.7)	163(38.1)			1.14(0.82,1.60)	0.43	0.81
		additive	GG	200(91.7)	245(92.5)	0.08	0.87			
			AA	18(8.3)	20(7.5)			0.87(0.41,1.84)	0.72	0.80
	rs2493134	codominant	CC	200(60.8)	244(57.0)	1.76	0.41			
			TC	111(33.7)	164(38.3)			1.20(0.86,1.66)	0.28	0.88
			TT	18(5.5)	20(4.7)			0.87(0.42,1.82)	0.28	0.88
		dominant	CC	200(60.8)	244(57.0)	1.10	0.30			
			TC+TT	129(39.2)	184(43.0)			1.10(0.79,1.51)	0.58	0.74
		recessive	CC+TC	311(94.5)	408(95.3)	0.25	0.74			
			TT	18(5.5)	20(4.7)			0.80(0.39,1.67)	0.56	0.83
		overdominant	CC+TT	218(66.3)	264(61.7)					
			TC	111(33.7)	164(38.3)			1.15(0.83,1.61)	0.41	0.74
		additive	CC	200(91.7)	244(92.4)	0.08	0.87			
			TT	18(8.3)	20(7.6)			0.88(0.42,1.85)	0.73	0.75
	rs2004776	codominant	TT	102(31.0)	116(27.1)	1.56	0.46			
			CT	166(50.5)	223(52.1)			1.46(0.73,2.91)	0.29	0.88
			CC	61(18.5)	89(20.8)			2.19(0.72,6.67)	0.17	0.88
		dominant	TT	102(31.0)	116(27.1)	1.38	0.26			
			CT+CC	227(69.0)	312(72.9)			1.21(0.85,1.72)	0.29	0.74

		recessive	TT+CT	268(81.5)	339(79.2)	0.59	0.46			
			CC	61(18.5)	89(20.8)			1.18(0.79,1.76)	0.43	0.83
		overdominant	TT+CC	163(49.5)	205(47.9)	0.20	0.66			
			CT	166(50.5)	223(52.1)			1.06(0.77,1.46)	0.73	0.64
		additive	TT	102(62.6)	116(56.6)	1.35	0.29			
			CC	61(37.4)	89(43.4)			1.35(0.84,2.15)	0.21	0.63
	rs2148582	codominantl	GG	200(60.8)	244(57.0)	1.76	0.41			
			AG	111(33.7)	164(38.3)			-	1	0.88
			AA	18(5.5)	20(4.7)			0.60(0.23,1.57)	0.30	0.88
		dominant	GG	200(60.8)	244(58.7)	1.10	0.30			
			AG+AA	129(39.2)	184(41.3)			1.10(0.79,1.51)	0.58	0.74
		recessive	GG+AG	311(94.5)	408(95.3)	0.25	0.74			
			AA	18(5.5)	20(4.7)			0.80(0.39,1.67)	0.56	0.83
		overdominant	GG+AA	218(66.3)	264(61.7)	1.69	0.20			
			AG	111(33.7)	164(38.3)			1.15 (0.83,1.61)	0.41	0.74
		additive	GG	200(91.7)	244(92.4)	0.08	0.87			
			AA	18(8.3)	20(7.6)			0.88(0.42,1.85)	0.73	0.75
	rs5046	codominant	GG	236(71.7)	311(72.7)	3.98	0.14			
			GA	89(27.1)	103(24.0)			1.17(0.62,2.18)	0.63	0.88
			AA	4(1.2)	14(3.3)			5.49(1.08,27.97)	0.04	0.88
		dominant	GG	236(71.7)	311(72.7)	0.78	0.81			
			GA+AA	93(28.3)	117(26.3)			1.01(0.70,10.41)	0.98	0.96
		recessive	GG+GA	325(98.8)	414(96.7)	3.39	0.09			
			AA	4(1.2)	14(3.3)			3.82(1.08,13.50)	0.07	0.71
		overdominant	GG+AA	240(72.9)	325(76.0)	0.88	0.36			
			GA	89(27.1)	103(24.0)			0.88(0.61,1.26)	0.48	0.74
		additive	GG	236(98.3)	311(96.1)	3.12	0.09			
			AA	4(1.7)	14(3.9)			3.47(0.98,12.36)	0.06	0.75
	rs3789679	codominant	GG	149(45.3)	200(46.7)	2.08	0.35			
			GA	135(41.0)	184(43.0)			1.35(0.76,2.40)	0.31	0.88
			AA	45(13.7)	44(10.3)			1.21(0.41,3.59)	0.73	0.88
		dominant	GG	149(45.3)	200(46.7)	0.16	0.71			
			GA+AA	180(54.7)	228(53.3)			0.94(0.68,1.29)	0.68	0.74
		recessive	GG+GA	284(86.3)	384(89.7)	2.07	0.17			
			AA	45(13.7)	44(10.3)			0.68(0.41,1.11)	0.12	0.71
		overdominant	GG+AA	194(59.0)	244(57.0)	0.29	0.60			
			GA	135(41.0)	184(43.0)			1.10(0.80,1.53)	0.55	0.64
		additive	GG	149()76.8	200(82.0)	1.78	0.19			
			AA	45(23.2)	44(18.0)			0.73(0.44,1.24)	0.24	0.63
ACE	*rs4316*	codominant	TT	132(40.1)	176(41.1)	1.73	0.42			
			CT	160(48.6)	192(44.9)			0.11(0.01,2.08)	0.14	0.93
			CC	37(11.2)	60(14.0)			0.35(0.01,22.93)	0.62	0.93
		dominant	TT	132(40.1)	176(41.1)	0.08	0.82			
			CT+CC	197(59.1)	252(58.9)			0.99(0.71,1.36)	0.93	0.74
		recessive	TT+CT	292(88.8)	368(86.0)	1.28	0.27			
			CC	37(11.2)	60(14.0)			1.15(0.71,1.87)	0.57	0.83
		overdominant	TT+CC	169(51.4)	236(55.1)	1.06	0.31			
			CT	160(48.6)	192(44.9)			0.93(0.67,1.28)	0.64	0.74
		additive	TT	132(78.1)	176(74.6)	0.67	0.48			
			CC	37(21.9)	60(25.4)			1.16(0.68,1.97)	0.58	0.75
	rs4343	codominant	AA	131(39.9)	174(40.7)	1.67	0.44			
			GA	161(48.9)	194(45.3)			8.84(0.44,177.92)	0.16	0.93
			GG	37(11.2)	60(14.0)			3.29(0.05,227.95)	0.58	0.93
		dominant	AA	131(39.9)	174(40.7)	0.05	0.82			
			GA+GG	198(60.1)	254(59.3)			1.01(0.73,1.40)	0.95	0.99
		recessive	AA+GA	292(88.8)	368(86.0)	1.28	0.27			
			GG	37(11.2)	60(14.0)			1.12(0.69,1.82)	0.65	0.83
		overdominant	AA+GG	168(51.1)	234(54.7)	0.97				
			GA	161(48.9)	194(45.3)			0.96(0.70,1.32)	0.81	0.94
		additive	AA	131(78.0)	174(74.4)	0.70	0.41			
			GG	37(22.0)	60(25.6)			1.16(0.68,1.97)	0.58	0.75
AGTR1	*rs5182*	codominant	TT	169(51.4)	242(56.5)	2.53	0.28			
			CT	137(41.6)	154(36.0)			0.59(0.36,0.96)	0.03	0.88
			CC	23(7.0)	32(7.5)			0.82(0.32,2.09)	0.68	0.88
		dominant	TT	169(51.4)	242(56.5)	2.01	0.16			
			CT+CC	160(48.6)	186(43.5)			0.88(0.64,1.21)	0.42	0.74
		recessive	TT+CT	306(83.0)	396(82.5)	0.07	0.89			
			CC	23(7.0)	32(7.5)			1.47(0.79,2.73)	0.23	0.83
		overdominant	TT+CC	192(58.4)	274(64.0)	2.52	0.11			
			CT	137(41.6)	154(36.0)			1.34(0.70,2.55)	0.38	0.88
		additive	TT	169(88.0)	242(88.3)	0.01	1			
			CC	23(12.0)	32(11.7)			1.34(0.70,2.55)	0.38	0.88
	rs1492100	codominant	TT	264(80.2)	340(79.4)	0.65	0.76			

			TA	63(19.2)	83(19.4)			1.03(0.64,1.64)	0.90	0.88
			AA	2(0.6)	5(1.2)			2.40(0.37,15.4)	0.36	0.88
		dominant	TT	264(80.2)	340(79.4)	0.08	0.86			
			TA+AA	65(19.8)	88(20.6)			1.20(0.81,1.78)	0.37	0.74
		recessive	TT+TA	327(99.4)	423(98.8)	0.64	0.48			
			AA	2(0.6)	5(1.2)			2.29(0.40,13.01)	0.35	0.83
		overdominant	TT	266(80.8)	345(80.6)	0.01	1			
			TT+AA	63(19.2)	83(19.4)			1.15(0.77,1.72)	0.50	0.74
		additive	TA	264(99.2)	340(98.6)	0.65	0.48			
			AA	2(0.8)	5(1.4)			2.25(0.40,12.60)	0.36	0.75
	rs5186	codominant	AA	295(89.7)	391(91.4)	0.63	0.73			
			AC	33(10.0)	36(8.4)			1.28(0.64,2.55)	0.76	0.97
			CC	1(0.3)	1(0.2)			1.71(0.07,41.97)	0.53	0.97
		dominant	AA	295(89.7)	391(91.4)	0.63	0.45			
			AC+CC	34(10.3)	37(8.6)			1.06(0.62,1.82)	0.83	0.96
		recessive	AA+AC	329(99.7)	426(99.8)	1.54	0.51			
			CC	1(0.3)	1(0.2)			-	1.00	1.00
		overdominant	AA+CC	296(90.0)	392(91.6)	0.59	0.45			
			AC	33(10.0)	36(8.4)			1.04(0.60,1.80)	0.89	0.99
		additive	AA	295(99.7)	391(99.7)	0.04	1			
			CC	1(0.3)	1(0.3)			1.97(0.12,33.22)	0.64	0.99
	rs275646	codominant	CC	281(85.4)	355(82.9)	1.02	0.60			
			TC	46(14.0)	71(16.6)			1.70(0.92,3.16)	0.09	0.88
			TT	2(0.6)	2(0.5)			1.27(0.11,14.93)	0.85	0.88
		dominant	CC	281(85.4)	355(82.9)	0.84	0.37			
			TC+TT	48(14.6)	73(17.1)			1.33(0.86,2.07)	0.20	0.74
		recessive	CC+TC	327(99.4)	426(99.5)	0.07	1.00			
			TT	2(0.6)	2(0.5)			1.76(0.21,14.55)	0.60	0.83
		overdominant	CC+TT	283(86.0)	357(83.4)	0.97	0.36			
			TC	46(14.0)	71(16.6)			1.31(0.84,2.05)	0.24	0.64
		additive	CC	281(99.3)	355(99.4)	0.06	1			
			TT	2(0.7)	2(0.6)			1.65(0.21,12.99)	0.64	0.73
	rs2933249	codominant	GG	252(76.6)	319(74.5)	0.48	0.82			
			GA	72(21.9)	101(23.6)			1.06(0.55,2.04)	0.86	0.97
			AA	5(1.5)	8(1.9)			0.66(0.10,4.42)	0.67	0.97
		dominant	GG	252(76.6)	319(74.5)	0.43	0.55			
			GA+AA	77(23.4)	109(25.5)			1.02(0.70,1.48)	0.93	0.99
		recessive	GG+GA	324(98.5)	420(98.1)	0.14	0.79			
			AA	5(1.5)	8(1.9)			1.09(0.30,3.90)	0.90	0.99
		overdominant	GG+AA	257(78.1)	327(76.4)	0.31	0.60			
			GA	72(21.9)	101(23.6)			1.01(0.69,1.48)	0.95	0.99
		additive	GG	252(98.0)	319(97.6)	0.17	0.78			
			AA	5(2.0)	8(2.4)			1.09(0.29,4.14)	0.90	0.99
	rs2638360	codominant	AA	268(81.5)	341(79.7)	1.32	0.53			
			GA	58(17.6)	79(18.4)			1.13(0.53,2.40)	0.76	0.97
			GG	3(0.9)	8(1.9)			3.00(0.35,25.84)	0.32	0.97
		dominant	AA	268(81.5)	341(79.7)	0.38	0.58			
			GA+GG	61(18.50)	87(20.30)			1.02(0.68,1.53)	0.94	0.99
		recessive	AA+GA	326(99.1)	420(98.1)	1.19	0.37			
			GG	3(0.9)	8(1.9)			1.48(0.35,6.25)	0.59	0.83
		overdominant	AA+GG	271(82.4)	349(81.6)	0.09	0.78			
			GA	58(17.6)	79(18.4)			0.98(0.65,1.49)	0.93	0.99
		additive	AA	211(98.6)	278(97.2)	1.11	0.37			
			GG	3(1.4)	8(2.8)			1.39(0.33,5.94)	0.65	0.75
CYP11B2	*rs6433*	codominant	TT	230(69.9)	299(69.9)	0.00	1			
			CT	89(27.1)	116(27.1)			0.81(0.54,1.22)	0.31	0.97
			CC	10(3.0)	13(3.0)			0.52(0.18,1.51)	0.23	0.97
		dominant	TT	230(69.9)	299(69.9)	0.00	1			
			CT+CC	99(30.1)	129(30.1)			0.94(0.66,1.33)	0.72	0.93
		recessive	TT+CT	319(97.0)	415(97.0)	0.00	1			
			CC	10(3.0)	13(3.0)			0.89(0.35,2.29)	0.81	0.91
		overdominant	TT+CC	240(82.9)	312(82.9)	0.00	1			
			CT	89(27.1)	116(27.1)			0.95(0.66,1.36)	0.78	0.87
		additive	TT	230(95.8)	299(95.8)	0.00	1			
			CC	10(4.2)	13(4.2)			0.86(0.32 ,2.29)	0.76	0.88
	rs3802228	codominant	AA	126(38.3)	186(43.5)	2.12	0.34			
			AG	164(49.8)	198(46.3)			0.69(0.28,1.67)	0.41	0.88
			GG	39(11.9)	44(10.3)			0.45(0.10,2.08)	0.31	0.88
		dominant	AA	126(38.3)	186(43.5)	2.04	0.16			
			AG+GG	203(61.7)	242(56.5)			0.78(0.56,1.08)	0.13	0.74
		recessive	AA+AG	290(98.1)	384(90.7)	0.47	0.56			
			GG	39(11.9)	44(10.3)			0.83(0.49,1.38)	0.46	0.83
		overdominant	AA+GG	165(50.2)	230(53.7)	0.96	0.34			
			AG	164(49.8)	198(46.3)			0.85(0.61,1.16)	0.30	0.64
		additive	AA	126(76.4)	186(80.9)	1.18	0.32			

			GG	39(23.6)	44(19.1)			0.77(0.44,1.35)	0.36	0.63
	rs1799998	codominant	AA	141(42.9)	204(47.7)	1.73	0.42			
			AG	156(47.4)	186(43.4)			1.13(0.47,2.74)	0.79	0.88
			GG	32(9.7)	38(8.9)			1.63(0.33,8.07)	0.55	0.88
		dominant	AA	141(42.9)	204(47.7)	1.73	0.21			
			AG+GG	188(51.7)	224(52.3)			0.80(0.58,1.10)	0.16	0.74
		recessive	AA+AG	297(90.3)	390(91.1)	0.16	0.71			
			GG	32(9.7)	38(8.9)			0.84 (0.49,1.47)	0.55	0.83
		overdominant	AA+GG	173(52.6)	242(56.6)	1.18	0.30			
			AG	156(47.4)	186(43.4)			0.84(0.61,1.16)	0.29	0.64
		additive	AA	141(81.5)	204(56.5)					
			GG	32(19.5)	38(43.5)	0.56	0.51	0.82(0.45,1.47)	0.50	0.63
LDLR	*rs688*	codominant	CC	247(75.1)	308(72.0)	1.99	0.37			
			CT	74(22.5)	113(26.4)			1.09(0.72,1.64)	0.69	0.88
			TT	8(2.4)	7(1.6)			0.61(0.16,2.23)	0.45	0.88
		dominant	CC	247(75.1)	308(72.0)	0.92	0.36			
			CT+TT	82(24.9)	120(28.0)			1.06(0.74,1.53)	0.76	0.74
		recessive	CC+CT	321(97.6)	421(98.4)	0.61	0.60			
			TT	8(2.4)	7(1.6)			0.66(0.20,2.20)	0.50	0.83
		overdominant	CC+TT	255(77.5)	315(73.6)	1.53	0.23			
			CT	74(22.5)	113(26.4)			1.11(0.76,1.61)	0.60	0.74
		additive	CC	247(96.9)	308(97.8)	0.46	0.60			
			TT	8(3.1)	7(2.2)			0.62(0.18,2.11)	0.44	0.75
LRP5	*rs638051*	codominant	AA	166(50.5)	126(50.5)	0.25	0.88			
			GA	137(41.6)	174(40.6)			0.99(0.68,1.44)	0.97	0.93
			GG	26(7.9)	38(8.9)			1.37(0.69,2.74)	0.37	0.93
		dominant	AA	166(50.5)	216(50.5)	0.00	1			
			GA+GG	163(49.5)	212(49.5)			1.20(0.87,1.65)	0.28	0.74
		recessive	AA+GA	303(92.1)	390(91.1)	0.23	0.69			
			GG	26(7.9)	38(8.9)			1.42(0.78,2.58)	0.26	0.83
		overdominant	AA+GG	192(58.4)	254(59.4)	0.08	0.82			
			GA	137(41.6)	174(40.6)			1.08(0.78,1.50)	0.63	0.74
		additive	AA	166(86.5)	216(85.0)	0.18	0.69			
			GG	26(13.5)	38(15.0)			1.49(0.79,2.81)	0.22	0.75
	rs556442	codominant	AA	175(53.2)	222(51.9)	1.42	0.50			
			GA	123(37.4)	174(40.6)			1.31(0.90,1.90)	0.16	0.88
			GG	31(9.4)	32(7.5)			0.93(0.49,1.78)	0.83	0.88
		dominant	AA	175(53.2)	222(51.9)	0.13	0.77			
			GA+GG	154(46.8)	206(48.1)			1.23(0.89,1.70)	0.20	0.74
		recessive	AA+GA	298(90.6)	396(92.5)	0.92	0.36			
			GG	31(9.4)	32(7.5)			0.79(0.44,1.41)	0.43	0.83
		overdominant	AA+GG	206(62.6)	254(59.4)	0.83	0.37			
			GA	123(37.4)	174(40.6)			1.34(0.96,1.87)	0.08	0.74
		additive	AA	175(85.0)	222(87.4)	0.58	0.50			
			GG	31(15.0)	32(12.6)			0.89(0.49,1.62)	0.71	0.75
LRP6	*rs10743980*	codominant	CC	184(55.9)	252(58.9)	0.88	0.65			
			TC	120(36.5)	149(34.8)			1.64(0.43,6.25)	0.47	0.88
			TT	25(7.6)	27(6.3)			10.18(0.49,212.25)	0.13	0.88
		dominant	CC	184(55.9)	252(58.9)	0.66	0.46			
			TC+TT	145(44.1)	176(41.1)			0.86(0.62,1.19)	0.36	0.74
		recessive	CC+TC	304(92.4)	401(93.7)	0.48	0.56			
			TT	25(7.6)	27(6.3)			0.70(0.39,1.38)	0.33	0.83
		overdominant	CC+TT	209(63.5)	279(65.2)	0.22	0.65			
			TC	120(36.5)	149(34.8)			0.93(0.67,1.29)	0.65	0.65
		additive	CC	180(94.3)	256(97.7)	3.68	0.06			
			TT	11(5.7)	6(2.3)			0.42(0.14,1.29)	0.13	0.68
	rs11054731	codominant	GG	147(44.7)	204(47.7)	2.01	0.36			
			AG	142(43.2)	185(43.2)			0.78(0.44,1.38)	0.40	0.88
			AA	40(12.1)	39(9.1)			0.46(0.16,1.31)	0.14	0.88
		dominant	GG	147(44.7)	204(47.7)	0.67	0.42			
			AG+AA	182(55.3)	224(52.3)			0.82(0.59,1.13)	0.22	0.74
		recessive	GG+AG	289(87.9)	389(90.9)	1.85	0.19			
			AA	40(12.1)	39(9.1)			0.70(0.42,1.17)	0.17	0.82
		overdominant	GG+AA	187(56.8)	243(56.8)	0.00	1			
			AG	142(43.2)	185(43.2)			0.94(0.68,1.29)	0.69	0.84
		additive	GG	147(78.6)	204(84.0)	2.01	0.17			
			AA	40(21.4)	39(16.0)			0.69(0.40,1.18)	0.18	0.64
	rs2417086	codominant	AA	183(55.6)	252(58.9)	1.38	0.50			
			GA	119(36.2)	149(34.8)			1.80(0.35,9.18)	0.48	0.88
			GG	27(8.2)	27(6.3)			8.49(0.36,200.13)	0.19	0.88
		dominant	AA	183(55.6)	252(58.9)	0.81	0.38			
			GA+GG	146(44.4)	176(41.1)			0.83(0.61,1.15)	0.27	0.74
		recessive	AA+GA	302(91.8)	401(93.7)	1.01	0.32			

			GG	27(8.2)	27(6.3)			0.69(0.37,1.28)	0.24	0.82
		overdominant	AA+GG	210(63.8)	279(65.2)	0.15	0.70			
			GA	119(36.2)	149(34.8)			0.91(0.66,.28)	0.61	0.84
		additive	GG	183(87.1)	252(90.3)	1.23	0.31			
			AA	27(12.9)	27(9.7)			0.67(0.35,1.28	0.22	0.63
	rs7136900	codominant	GG	278(84.5)	349(81.5)	1.14	0.58			
			GA	49(14.9)	76(17.8)			1.45(0.89,2.38)	0.14	0.97
			AA	2(0.6)	3(0.7)			0.64(0.09,4.63)	0.66	0.97
		dominant	GG	278(84.5)	349(81.5)	1.14	0.33			
			GA+AA	51(15.5)	79(18.5)			1.33 (0.86,2.06)	0.20	0.74
		recessive	GG+GA	327(99.4)	425(99.3)	0.03	1			
			AA	2(0.6)	3(0.7)			0.82(0.13,4.95)	0.82	0.91
		overdominant	GG+AA	280(85.1)	352(82.2)	1.11	0.32			
			GA	49(14.9)	76(17.8)			1.37(0.88,2.13)	0.17	0.74
		additive	GG	278(99.3)	349(99.1)	0.04	1			
			AA	2(0.7)	3(0.9)			0.76(0.12,4.66)	0.70	0.75
	rs12823243	codominant	AA	177(53.8)	249(58.2)	3.05	0.23			
			TA	122(37.1)	153(35.7)			0.36(0.11,1.17)	0.09	0.88
			TT	30(9.1)	26(6.1)			0.01(0.01,0.30)	0.01	0.88
		dominant	AA	177(53.8)	249(58.2)	1.45	0.24			
			TA+TT	152(46.2)	179(41.8)			0.78(0.57,1.08)	0.13	0.74
		recessive	AA+TA	299(90.9)	402(93.9)	2.52	0.12			
			TT	30(9.1)	26(6.1)			0.54(0.29,0.99)	0.04	0.71
		overdominant	AA+TT	207(62.9)	275(64.3)	0.14	0.76			
			TA	122(37.1)	153(35.7)			0.92(0.66,1.28)	0.63	0.86
		additive	AA	177(85.5)	249(90.5)	2.92				
			TT	30(14.5)	26(9.5)			0.53(0.28,1.00)	0.05	0.63
PPARG	*rs3856806*	codominant	CC	192(58.4)	282(65.9)	5.38	0.07			
			CT	118(35.8)	131(30.6)			0.79(0.52,1.20)	0.27	0.88
			TT	19(5.8)	15(3.5)			0.45(0.10,2.09)	0.13	0.88
		dominant	CC	192(58.4)	282(65.9)	4.51	0.03			
			CT+TT	137(41.6)	146(34.1)			0.70(0.51,0.97)	0.03	0.74
		recessive	CC+CT	310(94.2)	413(96.5)	2.24	0.16			
			TT	19(5.8)	15(3.5)			0.58(0.27,1.23)	0.16	0.82
		overdominant	CC+TT	211(64.2)	297(69.4)	2.33	0.14			
			CT	118(35.8)	131(30.6)			0.77(0.55,1.07)	0.12	0.64
		additive	CC	192(99.1)	282(99.5)	3.09	0.10			
			TT	19(0.9)	15(0.5)			0.52(0.24,1.15)	0.11	0.63
	rs1175543	codominant	AA	105(31.9)	140(32.7)	0.97	0,98			
			AG	159(48.3)	205(47.9)			0.94(0.61,1.45)	0.72	0.97
			GG	65(19.8)	83(19.4)			0.98(0.54,1.76)	0.94	0.97
		dominant	AA	105(31.9)	140(32.7)	0.05	0.88			
			AG+GG	224(68.1)	288(67.3)			0.87(0.62,1.22)	0.42	0.74
		recessive	AA+AG	264(80.2)	345(80.6)	0.02	0.93			
			GG	65(19.8)	83(19.4)			0.89(0.60,1.33)	0.57	0.91
		overdominant	AA+GG	170(51.7)	223(52.1)	0.01	0.94			
			AG	159(48.3)	205(47.9)			0.95(0.69,1.31)	0.76	0.84
		additive	AA	105(61.8)	140(62.8)	0.04	0.92			
			GG	65(38.2)	83(37.2)			0.84(0.52,1.35)	0.47	0.75
	rs2972164	codominant	CC	269(81.8)	352(82.2)	0.03	0.98			
			TC	57(17.3)	72(16.8)			0.66(0.40,1.08)	0.10	0.97
			TT	3(0.9)	4(1.0)			1.19(0.19,7.40)	0.85	0.97
		dominant	CC	269(81.8)	352(82.2)	0.03	0.92			
			TC+TT	60(18.2)	76(17.8)			0.82(0.54,1.25)	0.35	0.74
		recessive	CC+TC	326(99.1)	424(99.0)	0.01	1.00			
			TT	3(0.9)	4(1.0)			1.19(0.23,6.19)	0.84	0.91
		overdominant	CC+TT	272(82.7)	356(73.2)	0.03	0.92			
			TC	57(17.3)	72(16.8)			0.80(0.53,1.23)	0.32	0.74
		additive	CC	269(98.9)	352(98.9)	0.01	0.64			
			TT	3(1.1)	4(1.1)			1.22(0.23,6.49)	0.82	0.75
	rs13433696	codominant	GG	135(41.0)	194(45.3)	1.69	0.44			
			GA	150(45.6)	186(43.5)			0.76(0.44,1.31)	0.32	0.88
			AA	44(13.4)	48(11.2)			0.88(0.36,2.12)	0.77	0.88
		dominant	GG	135(41.0)	194(45.3)	1.40	0.27			
			GA+AA	194(59.0)	234(54.7)			0.73(0.53,1.01)	0.06	0.74
		recessive	GG+GA	285(86.6)	380(88.8)	0.81	0.37			
			AA	44(13.4)	48(11.2)			0.79(0.49,1.30)	0.37	0.83
		overdominant	GG+AA	179(54.4)	242(56.5)	0.34	0.61			
			GA	150(45.6)	186(43.5)			0.81(0.59,1.11)	0.19	0.64
		additive	GG	135(75.4)	194(80.2)	1.36	0.28			
			AA	44(24.6)	48(19.8)			0.69(0.41,1.18)	0.16	0.63
	rs9817428	codominant	AA	107(32.5)	127(29.7)	1.16	0.56			
			CA	151(45.9)	213(49.8)			1.02(0.59,1.77)	0.95	0.93
			CC	71(21.6)	88(20.6)			0.89(0.41,1.92)	0.76	0.93
		dominant	AA	107(32.5)	127(29.7)	0.71	0.43			

		CA+CC	222(67.5)	301(70.3)			1.17(0.83,1.65)	0.37	0.74
	recessive	AA+CA	258(78.4)	340(79.4)	0.12	0.79			
		CC	71(21.6)	88(20.6)			1.11(0.76,1.64)	0.59	0.91
	overdominant	AA+CC	178(54.1)	215(50.2)	1.12	0.31			
		CA	151(45.9)	213(49.8)			1.07(0.77,1.47)	0.70	0.83
	additive	AA	107(60.1)	127(59.1)	0.04	0.84			
		CC	71(39.9)	88(40.9)			1.29(0.81,2.04)	0.28	0.83
rs12631819	codominant	GG	129(39.2)	176(41.1)	0.57	0.74			
		GT	157(47.7)	203(47.5)			0.93(0.56,1.55)	0.79	0.93
		TT	43(13.1)	49(11.4)			0.78(0.35,1.75)	0.54	0.93
	dominant	GG	129(39.2)	176(41.1)	0.28	0.60			
		GT+TT	200(60.8)	252(58.9)			0.78(0.57,1.08)	0.14	0.74
	rcsessive	GG+GT	286(86.9)	379(88.6)	0.46	0.50			
		TT	43(13.1)	49(11.4)			0.87(0.54,1.42)	0.59	0.83
	overdominant	GG+TT	172(52.3)	225(52.5)	0.01	0.94			
		GT	157(47.7)	203(47.5)			0.84(0.61,1.15)	0.27	0.74
	additive	GG	129(75.0)	176(78.2)	0.57	0.47			
		TT	43(25.0)	49(21.8)			0.79(0.46,1.35)	0.39	0.75

Note: Chi-square tests were used to analyze the distribution of genotypes between case controls (p_1), and multifactorial logistic regression (OR_2, p_3) was used to analyze the influence of genotypes on disease, p_{adj} was adjusted by FDR-BH correction.

Table S10-1. The D′ and R^2 value of Linkage Disequilibrium-block 1

D'/R²	rs12823243	rs2972164	rs9817428	rs12631819	rs13433696	rs11054731	rs10743980	rs7136900	rs1175543	rs3856806
rs12823243		0.288/0.003	0.044/0.001	0.004/0	0.078/0.001	0.922/0.619	0.963/0.884	0.467/0.062	0.139/0.005	0.001/0
rs2972164			1/0.127	1/0.058	0.958/0.05	0.136/0.001	0.273/0.003	0.032/0.001	0.429/0.015	0.487/0.007
rs9817428				1/0.46	0.984/0.415	0.048/0.001	0.056/0.001	0.03/0	0.657/0.273	0.701/0.107
rs12631819					0.673/0.423	0.02/0	0.008/0	0/0	0.476/0/164	0.397/0.074
rs13433696						0.08/0.002	0.082/0.001	0.015/0	0.557/0.21	0.787/0.313
rs11054731							0.996/0.688	0.521/0.056	0.141/0.007	0.031/0
rs10743980								0.483/0.07	0.18/0.008	0.002/0
rs7136900									0.13/0.001	0.035/0
rs1175543										0.416/0.059
rs3856806										

Table S10-2. The D′ and R^2 value of Linkage Disequilibrium-block 2

D'/R²	rs4316	rs4343	rs4461142
rs4316		0.997/0.986	0.701/0.355
rs4343			0.708/0.365
rs4461142			

Table S10-3. The D′ and R^2 value of Linkage Disequilibrium-block 3

D'/R²	*rs638051*	*rs556442*
rs638051		0.159/0.024
rs556442		

Table S10-4. The D′ and R^2 value of Linkage Disequilibrium-block 4

D'/R²	*rs3802228*	*rs6433*	*rs1799998*
rs3802228		0.975/0.101	0.997/0.866
rs6433			0.97/0.087
rs1799998			

Table S10-5. The D′ and R^2 value of Linkage Disequilibrium-block 5

D'/R²	*rs2933249*	*rs2638360*	*rs1492100*	*rs5182*	*rs5186*	*rs275646*
rs2933249		0.895/0.621	1/0.018	0.513/0.11	0.147/0.007	0.473/0.003
rs2638360			1/0.014	0.653/0.139	1/0.006	0.426/0.002
rs1492100				0.194/0.012	0.011/0	0.375/0.107
rs5182					1/0.141	0.96/0.23
rs5186						0.595/0.002
rs275646						

Table S10-6. The D′ and R^2 value of Linkage Disequilibrium-block 6

D'/R^2	*rs699*	*rs2004776*	*rs2493134*	*rs3789679*	*rs2148582*	*rs2417086*	*rs5046*
rs699		1/0.36	1/0.996	1/0.147	1/0.996	0.035/0.001	1/0.53
rs2004776			1/0.361	1/0.408	1/0.361	0.028/0	1/0.148
rs2493134				1/0.147	1/1	0.033/0.001	1/0.054
rs3789679					1/0.147	0.134/0.003	1/0.087
rs2148582						0.033/0.001	1/0.054
rs2417086							0.023/0
rs5046							
